# A novel remitting leukodystrophy associated with a variant in *FBP2*

**DOI:** 10.1093/braincomms/fcab036

**Published:** 2021-03-11

**Authors:** Agnieszka Gizak, Susann Diegmann, Steffi Dreha-Kulaczewski, Janusz Wiśniewski, Przemysław Duda, Andreas Ohlenbusch, Brenda Huppke, Marco Henneke, Wolfgang Höhne, Janine Altmüller, Holger Thiele, Peter Nürnberg, Dariusz Rakus, Jutta Gärtner, Peter Huppke

**Affiliations:** 1 Department of Molecular Physiology and Neurobiology, University of Wrocław, 50-335 Wrocław, Poland; 2 Department of Pediatrics and Pediatric Neurology, University Medical Center Göttingen, Georg August University, 37075 Göttingen, Germany; 3 Department of Neuropediatrics, Jena University Hospital, 07747 Jena, Germany; 4 Cologne Center for Genomics (CCG) and Center for Molecular Medicine Cologne (CMMC), University of Cologne, 50931 Cologne, Germany

**Keywords:** leukodystrophy, remitting, muscle fructose 1,6-bisphosphatase

## Abstract

Leukodystrophies are genetic disorders of cerebral white matter that almost exclusively have a progressive disease course. We became aware of three members of a family with a disorder characterized by a sudden loss of all previously acquired abilities around 1 year of age followed by almost complete recovery within 2 years. Cerebral MRI and myelin sensitive imaging showed a pronounced demyelination that progressed for several months despite signs of clinical improvement and was followed by remyelination. Exome sequencing did not-identify any mutations in known leukodystrophy genes but revealed a heterozygous variant in the *FBP2* gene, c.343G>A, p. Val115Met, shared by the affected family members. Cerebral MRI of other family members demonstrated similar white matter abnormalities in all carriers of the variant in *FBP2*. The *FBP2* gene codes for muscle fructose 1,6-bisphosphatase, an enzyme involved in gluconeogenesis that is highly expressed in brain tissue. Biochemical analysis showed that the variant has a dominant negative effect on enzymatic activity, substrate affinity, cooperativity and thermal stability. Moreover, it also affects the non-canonical functions of muscle fructose 1,6-bisphosphatase involved in mitochondrial protection and regulation of several nuclear processes. In patients’ fibroblasts, muscle fructose 1,6-bisphosphatase shows no colocalization with mitochondria and nuclei leading to increased reactive oxygen species production and a disturbed mitochondrial network. In conclusion, the results of this study indicate that the variant in *FBP2* disturbs cerebral energy metabolism and is associated with a novel remitting leukodystrophy.

## Teaser

Gizak et al. report on a novel remitting leukodystrophy associated with a variant in the *FBP2* gene coding for muscle fructose 1,6-bisphosphatase. The variant has a dominant negative effect not only on enzymatic properties and stability but also on non-canonical functions important for mitochondrial protection and regulation of nuclear processes.

## Graphical Abstract

**Graphical Abstract:**
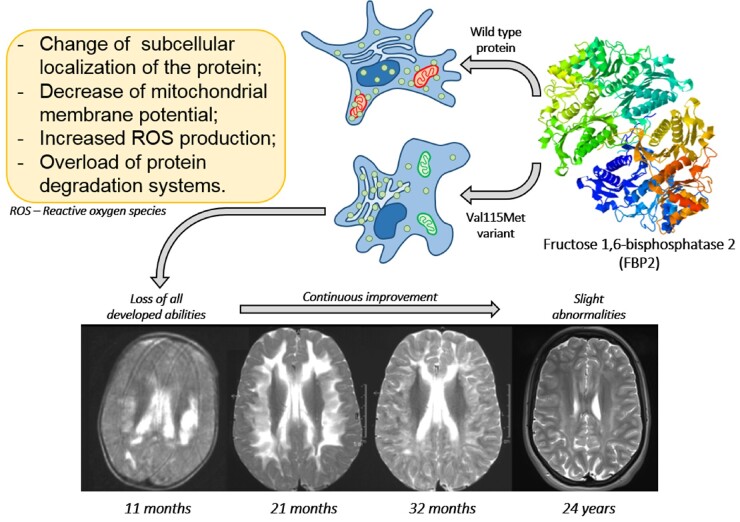

## Introduction

Leukodystrophies are a group of genetic disorders with primary involvement of the central nervous system white matter.^1^ Most of these disorders have a progressive neurodegenerative course often leading to premature death. Four leukodystrophies with a remitting course have, however, recently been described: (i) Patients with variants in the *TMEM63A* gene, coding for a mechanosensitive ion channel, present with congenital nystagmus and motor delay in infancy associated with hypomyelination of the CNS.^2^ During infancy the myelin deficit resolves, and the clinical course is favourable. (ii) Variants in the *SLC13A3* gene, encoding the plasma membrane Na^+^/dicarboxylate cotransporter 3, have been associated with a reversible leukoencephalopathy during febrile illness.^3^ Clinical recovery was observed within days of the crisis and MRI changes normalized. (iii) Recessive mutations in *GLIALCAM* cause a classical megalencephalic leukoencephalopathy with subcortical cysts. Autosomal dominant mutations in the same gene, however, have been associated with a remitting phenotype.^4^ (iv) Finally, two families with mutations in *FDX2,* coding for a protein involved in mitochondrial [Fe-S] protein assembly, have recently been described in whom partial resolution of the leukoencephalopathy was reported but the clinical course remained progressive.^5^

In this article, we describe a family with a disorder that can also be classified as a remitting leukodystrophy. Affected family members presented with very pronounced white matter abnormalities that almost completely resolved over time, accompanied by remarkable clinical improvement. A missense variant of the *FBP2* gene coding for the muscle fructose 1,6-bisphosphatase (FBP2) segregates with the disorder. FBP is a regulatory enzyme of gluconeogenesis that catalyzes the hydrolysis of fructose-1,6-bisphosphate (F-1,6-BP) to fructose-6-phosphate (F-6-P) and inorganic phosphate. In contrast to the liver isozyme, fructose 1,6-bisphoshatase (FBP1), which regulates glucose production in the liver, kidney and jejunum, FBP2 expression is not restricted to gluconeogenic tissues but is ubiquitous.^6^ Crystallographic studies have demonstrated that FBP2 is a homotetrameric protein which may exist in two quaternary conformations: an active cross-like structure R-state, and an inactive T-state.^7^ Allosteric inhibitors such as adenosine monophosphate (AMP) and nicotinamide adenine dinucleotide (NAD^+^) stabilize the tetrameric conformation of FBP2 and induce a shift towards the T-state.^7^ The capability of FBP2 to adopt various quaternary conformations determines its potential to interact with different cellular binding partners and assume different cellular roles depending on the metabolic and physiological conditions.

During the last decade it has been shown that FBP2 has also acquired additional functions as a moonlighting protein. The non-canonical functions include FBP2 association with mitochondrial proteins in GSK3β- and calcium-dependent manners, thereby making the protein instrumental in the protection of organelles against stress stimuli.^8^ Furthermore, FBP2 is known to localize in nuclei where it is involved in the regulation of cell mortality and survival. Biochemical and clinical evidence suggests that the variant of FBP2 detected in the family described in this article acts in a dominant negative fashion, affecting both enzymatic activity and the non-canonical functions of the enzyme.

## Materials and methods

DNA samples were obtained following informed consent according to the Declaration of Helsinki and approval by the ethic commission from the University Medical Center Göttingen, Göttingen (approval number 2516). Fibroblasts from affected individuals used in this study were obtained from skin biopsies previously sampled for routine diagnostic testing.

### Proton magnetic resonance spectroscopy

Our multimodal MR-protocol included single-voxel proton Magnetic Resonance Spectroscopy (MRS). Proton MR spectra (64 accumulations) were acquired with use of a STEAM localization sequence with repetition time/echo time/mixing time = 6000/20/10 ms as described.^9^ Studies older than 15 years were conducted at 2T (Magnetom Vision; Siemens Medical Solutions, Erlangen, Germany) all others at 3T (Magnetom Trio, TIM TRIO and Prisma Fit; Siemens Healthcare). The 4.85 ml (2T) and 4.1 ml (3T) volumes-of-interest were placed within the parieto-occipital white matter (left or right) covering structural lesions. Absolute concentrations of lactate were determined by LCModel.^10^ Normal ranges were taken from Pouwels et al.^11^ and our own data base.

### Magnetization transfer-imaging

Whole brain magnetization transfer (MT)-imaging, as another part of our myelin specific MR-imaging protocol, was performed at 3T (Magnetom TIM TRIO and Prisma Fit) using a 3D FLASH sequence with 1.25 mm isotropic resolution and 240 mm field-of-view. MT contrast was imposed upon a proton density-weighted reference (TR/TE/*α*  =  25/4.9 ms/5°, using partial acquisition techniques) by applying a 12.8 ms Gaussian MT-pulse of 540° nominal flip angle 2.2 kHz off resonance prior to excitation. By means of a second T1-weighted reference (TR/*α* = 11 ms/15°, 1.5 min), maps of the percentage MT saturation (MTsat) were calculated and a semi-quantitative MT-parameter as described in Helms et al.^12^ Data processing was scripted using the routines of the FSL 4.1 software library of the Center for Functional Magnetic Resonance Imaging of the Brain (FMRIB, Oxford, UK, www.fmrib.ox.ac.uk/fsl 17 March 2021, date last accessed).^13^^,^^14^ The blue-grey-red-yellow colour scale of the MTsat maps covered a range from –0.1 pu (light blue; lower limit of the CSF mode) to 1.2 pu (grey, grey matter) to 2.5 pu (yellow; fully myelinated white matter).^15^

### Sequencing of mitochondrial DNA

Genomic DNA was extracted from peripheral blood from patient III.5 using standard techniques. Two overlapping mitochondrial DNA (mtDNA) fragments of 11 kb and 9 kb encompassing the entire mitochondrial genome were generated with the Expand Long Template PCR System (Roche, Mannheim, Germany) according to the recommendations of the manufacturer. Sanger sequencing was performed using the BigDyeTM Terminator v3.1 Cycle Sequencing chemistry on an Applied Biosystems 3500 Genetic Analyzer platform (Applied Biosystems, Darmstadt, Germany). Analysed sequences were compared with reference NCBI data, accession number NC_012920.1. Primer sequences as well as PCR and sequencing conditions are available on request. Mitochondrial disease due to a large-scale mitochondrial DNA rearrangement was excluded by long-range PCR using the Expand Long Template PCR System (Roche, Mannheim, Germany). DNA from blood and urine epithelial cells was extracted using the NucleoSpin Blood DNA Kit (Machery-Nagel, Düren, Germany) according to the manufacturer’s protocol. All samples were collected on the same day. Long-range PCR using different pairs of primers generated overlapping mtDNA fragments covering the entire mitochondrial genome of 16 569 bp (GenBank NC_012920.1). Primer sequences as well as PCR conditions are available on request.

### Whole exome sequencing

**Figure 1:**
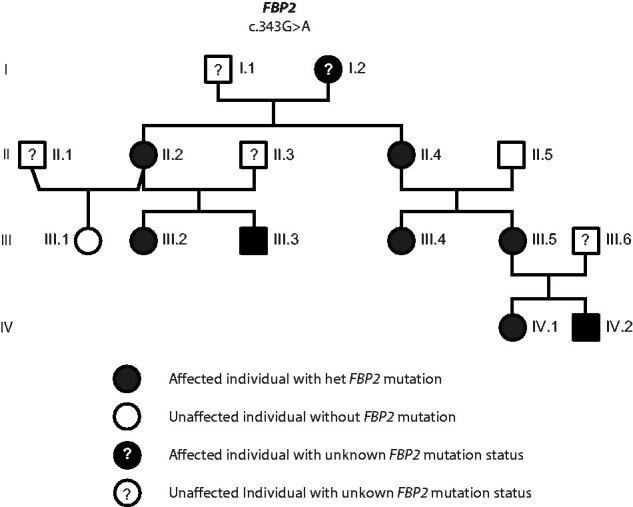
**Pedigree and segregation analysis of the affected Family.** Nomenclature is according to GenBank accession number NM_003837.2. Case reports on III.2, III.5 and IV.1 can be found in the results section. I.2, female, was born in 1948 and developed epilepsy and schizophrenia during her youth. She has never learnt a profession and lives in a shelter home. Her two children, II.2 II.4, grew up in an orphanage in the German Democratic Republic because she was incapable of looking after her. No information is available for the first 5 years of their life. Patient II2 developed severe anorexia as a teenager, which currently at age 47 is still present and prevents her from working in her profession as a nurse. II.4, developed epilepsy at age 4 years, and still now requires anticonvulsive medication at age 46. She works as a cleaner. III.3, aged 26 years, has a history of psychosis and drug addiction. He refused cerebral MRI investigation. III.4 developed epilepsy at age 9 years following a presumed viral infection with high fever. Development up to this point of time was normal with no signs of a metabolic crisis. She was treated with oxcarbazepine but continued to have two brief (2–3 min) focal seizures a month until age 13 years. At last presentation at age 14.5 years, she was seizure free and visiting a regular school. IV.2, now aged 6 years, has mildly delayed speech development and an attention deficit hyperactivity disorder. Family members I.1, II.1, II.3, II5 and III.1 have no known neurological or psychiatric disorders.

Whole exome sequencing was performed on DNA from ethylenediaminetetraacetic acid blood of six affected family members (II.2, II.4, III.2, III.4, III.5, IV.1 and Fig. 1). Each Sequencing barcoded library was prepared using the Agilent SureSelect V6 enrichment Kit. Purified and quantified library pool was subsequently sequenced on an Illumina HighSeq 4000 sequencing instrument (Illumina, San Diego, CA, USA) using a paired end 2x 75 bp to 2x 101 bp protocol and an allocation of 6 exomes per lane. Data processing, analysis and filtering were performed using the ‚Varbank 2’ GUI and pipeline version 3.4/3.5 (CCG, University of Cologne, Germany). Reads were mapped to the human genome reference build GRCh38 using the BWA-MEM alignment algorithm. GATK HaplotypeCaller, Samtools mpileup and Platypus variation calls were filtered for high-quality (QD > 5; ARF > 0.25; MQ > 50; FS < 40; MQRankSum>–5; ReadPosRankSum>–5; passed VQSR filter) rare (MAF ≤ 0.005 based on the maximum observed population allele frequency in gnomAD v2) variants, predicted to modify a protein sequence or to impair splicing and implicated by reduced maximum entropy scores (MaxEntScan).^16–18^

For detection of the common genetic cause of all affected family members the dataset was filtered for rare common genetic variants. False positive and irrelevant variants were further reduced by taking advantage of our InHouseDB. The pathogenicity of all received variants was evaluated using several *in silico* analysis tools by the dbNSFP/dbscSNV version 3.4 database.

Validation of variant of interest in *FBP2* as well as co-segregation analysis within the family was performed using Sanger sequencing.

### FBP2 expression, mutagenesis and fluorescent labelling

Human FBP2 mutant V115M was created using the procedure described earlier. The following primer pairs were used: FW: AAG AGG GGG AAA TAC ATG GTC TGC TTT GAC C; RV: GGT CAA AGC AGA CCA TGT ATT TCC CCC TCT T-3′. Sequences were confirmed by DNA sequencing using T7 promoter and T7 terminator primers.

WT-FBP2 and V115M-FBP2 were isolated according to Wisniewski et al.^9^ Both FBP2 variants were labelled using fluorescein isothiocyanate (FITC), as described previously.^19^

### Enzyme activity measurements

FBP2 activity was measured at pH 7.5 and 37°C using a glucose 6-phosphate isomerase—glucose 6-phosphate dehydrogenase coupled assay and the reduction of nicotinamide adenine dinucleotide phosphate (NADP^+^) to NADPH was monitored spectrophotometrically at 340 nm.^20^ Parameters were estimated in MATLAB’s curve fitting tool, using nonlinear least squares regression with trust-region algorithm and bisquare robust fitting.

FBP2 inhibition by AMP was measured in triplicate for each concentration of the ligand.

### Protein melting assay

Protein precipitation was assessed by measurement of light scattering at 600 nm wavelength using an Agilent 8453 UV–Vis spectrophotometer with 10 mm light path length. The measurements were conducted at temperatures ranging from 35°C to 80°C in 2°C steps. Samples were equilibrated for 2 min before each measurement. To avoid sedimentation, the samples were stirred at 200 rev min^−1^. Buffer and protein concentration were the same as in the enzymatic activity measurements. 300 µM AMP was added for T-state measurements. To determine the melting temperatures, derivative curves of absorbance versus temperature were calculated. The maxima of the derivative curves correspond to the melting temperatures.

### Cell culturing

Fibroblasts were cultured as we described before.^21^

### Fluorescent *in situ* hybridization (FISH)

FISH was performed as described in Ref.22 The following oligonucleotides complementary to human messenger RNA (mRNA) sequences were used: WT-FBP2 (5′-(Cyanine3) CCT CAG GGA ATT TCT TTT TCT GCA CAT ATC CAG TGG TGG C-3′), V115M-FBP2 (5′-(Cyanine5) CCA GTG GGT CAA AGC ACA TCA CGT ATT TCC CCC GCT TCT C-3′). All the oligonucleotides were synthesized by Merck KGaA. In controls, the oligonucleotide probes were omitted.

### Immunocytochemistry

Cells growing on coverslips were fixed in 4% paraformaldehyde, permeabilized with 0.1% Triton X-100 in phosphate-buffered saline (PBS) and incubated with 3% bovine serum albumin in PBS to reduce nonspecific binding of antibodies. Then they were incubated with the respective primary antibody: Mouse monoclonal to PSMC6 (1:500, Abcam, Cambridge, UK, ab22639), mouse monoclonal to LAMP-1 (1:500, Santa Cruz Biotechnology, Dallas, TX, USA, sc-20011) and rabbit polyclonal to FBP (1:500, purified and characterized as described in Ref.23).

The primary antibodies were then detected using fluorophore-labelled secondary antibodies: Goat polyclonal to mouse IgG (AlexaFluor488) (1:1000; Abcam, Cambridge, UK, ab150113), goat polyclonal to mouse IgG (AlexaFluor633) (1:1000; ThermoFisher Scientific, Waltham, MA, USA, a21050), goat polyclonal to rabbit IgG (AlexaFluor488) (1:1000; ThermoFisher Scientific, Waltham, MA, USA, a11034), goat polyclonal to rabbit IgG (AlexaFluor633) (1:1000; ThermoFisher Scientific, Waltham, MA, USA, a21070). To visualize mitochondria, the cells were counterstained with MitoTracker Deep Red (ThermoFisher). In the controls, the primary antibodies were omitted.

Images were acquired using FV-1000 confocal microscope (Olympus) with ×60 (oil, Plan SApo, NA = 1.35) objective using the Sequential scan option. The quantification of fluorescence signal was performed using the ImageJ software.^24^ The cells were marked and the mean fluorescence within the marked areas was measured. The measurements were taken from at least 50 cells from randomly selected areas. For the analysis of FBP2 colocalization with mitochondria, endoplasmic reticulum and PSMC6 the Manders’ coefficient was determined using the ImageJ software24. The coefficient varies from 0 (no colocalization) to 1 (100% of colocalization). It was determined as the ratio of summed intensities of pixels from the FBP2 channel for which the intensity in the mitochondrial/endoplasmic/PSMC6 channel was above zero, to the total intensity in the FBP2 channel. The measurements were taken from at least 18 randomly selected areas. The experiment was repeated in triplicate.

In all the fluorescence measurement experiments, *n* stands for number of cells. Results are expressed as a mean and standard deviation. If not stated otherwise, data were checked for normality using the Shapiro–Wilk test, for equality of variations using the F-test and for the evaluation of statistical significance the two-tailed Student’s *T*-test was used. For results deviating from normal distribution the Mann–Whitney’s U-test was used. A probability of *P* < 0.05 was considered to represent a significant difference. All the experiments were done at least in triplicate.

### Measurements of cellular reactive oxygen species (ROS) production and mitochondrial polarization

Cellular ROS production was measured using dihydrofluorescein diacetate (H2DCF-AC) as described in Ref.^25^ Briefly, live cells were loaded with 5 μM H2DCF-AC (20 min, 37°C), rinsed with Hank’s Balanced Salt Solution and mounted on slides. The fluorescence of the dye was excited at 488 nm for 500 ms. Mitochondrial ROS production was determined by MitoTracker Red CM-H2XRos (ThermoFisher) that fluoresces only upon oxidation.

Loss of the mitochondrial membrane potential was detected using JC-1 fluorescent dye (Mitochondrial Permeability Transition Detection Kit, AbD Serotec) according to the manufacturer’s instruction. Polarized mitochondria accumulate more of the dye and are red. In cells with depolarized mitochondria, most of the dye is dispersed in cytoplasm and has green fluorescence. The ratio of red to green fluorescence reflects the degree of the mitochondrial membrane polarization. The JC-1 dye was excited at 488 nm for 500 ms and the emission was observed using a long pass filter, which allowed for simultaneous observation of green (monomers) and red (aggregates) fluorescence. The state of the mitochondria polarization is presented as the red/green fluorescence intensity ratio, where reduction of the mitochondrial potential is indicated by a reduction in the ratio. The quantification of the fluorescent signals was performed and analysed as described above (‘Immunocytochemistry’). The measurements were taken from at least 50 cells from randomly selected areas. The experiments were repeated in duplicate.

### Protein delivery to fibroblasts and assessment of ERAD activity

Healthy fibroblasts were grown on coverslips. FITC-labelled FBP2 forms were delivered into the cells using CHARIOT Protein Delivery Reagent (Active Motif) as we described before.^19^ Then the cells were counterstained with MitoTracker Deep Red FM or with ER-Tracker Blue White (ThermoFisher) and examined under confocal microscope; in the images, they are shown in red. To test the endoplasmic reticulum-associated degradation of the V115M-FBP2 mutant, healthy fibroblasts were pre-incubated for 1 h with 8 µM Eeyarestatin, the inhibitor of ERAD (endoplasmic reticulum-associated protein degradation) and transfected with the mutant form of FBP2 in the presence of the inhibitor.

### Co-immunoprecipitation

In the co-immunoprecipitation experiment, the cell lysates obtained from cell cultures were used. Briefly, the lysates (100 µg protein) were mixed and incubated overnight at 4°C with 50 µg/ml of antibodies against ubiquitin (Lys48-specific, Merck KGaA, Darmstadt, Germany, 05–1307). The complexes were precipitated using 200 µl/ml of the Protein G Agarose (Merck). The precipitates were centrifuged at 4000× g for 2 min and washed with PBS. In control reactions, the precipitating antibodies were omitted. The precipitates were then resuspended in the Laemmli’s buffer, resolved by SDS–PAGE and Western Blot analyses were performed with the use of primary antibodies detecting FBP (isolated and purified as described in Ref. 23).

### Data availability

The data that support the findings of this study are available from the corresponding author, upon reasonable request.

## Results

### Case reports

**Figure 2:**
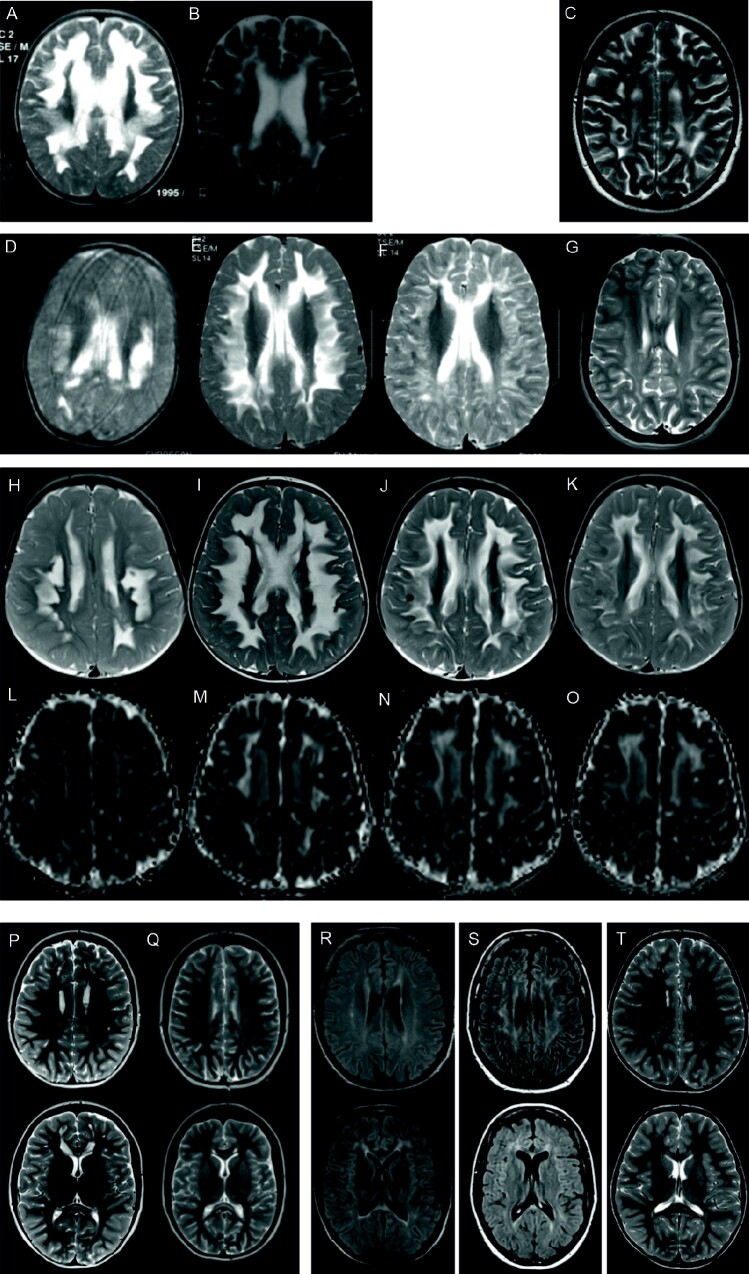
**MR imaging of the family members.** (**A–K**) Axial MR images of the patients with the remitting leukodystrophy. (**A–C**) T2 weighted images of patient III.2 at age 14 months, 22 months and 20 years. (**D–G**) T2 weighted images of patient III.5 at age 11 months, 21 months, 32 months and 24 years. (**H–K**) T2 weighted images of patient IV.1 at age 13, 19, 25 and 29 months. (**L–O**) Apparent diffusion coefficient of patient IV.1 at corresponding time points. (**P** and **Q**) T2 weighted images of patient III.4 at age 10 in **P** and 15 years in **Q** with residual WM lesions, pronounced frontally and in the genu corpus callosum. (**R**) Fluid-attenuated inversion recovery (FLAIR) images of patient II.2 at age 38 years revealing frontally pronounced residual WM lesion. (**S**) FLAIR images of patient II.4 at age 46 years with similar residual pattern and global atrophy. (**T**) T2 weighted images of patient IV.2 at age 4 years. Of note are the subtle WM hyperintensities in genu and splenium, comparable to patient III.4, II.2, II.4, although no metabolic crisis occurred.

The four-generation pedigree is shown in Fig. 1. Case reports and description of the MRI findings of the family members who did not present with a crisis at age 1 year are included in Figs. 1 and 2, respectively.

### Case reports of the three patients with a remitting leukoencephalopathy in infancy

Patient III.2, female, was born at term after an uneventful pregnancy. Psychomotor development was normal until age 13 months when, during a bronchitis episode with fever, she developed a paresis of the left leg followed within days by the loss of almost all abilities. At presentation to hospital she was irritable, socially withdrawn, unable to sit, crawl or stand and displayed only minimal motor activity. Significant feeding difficulties made tube feeding necessary for 4 weeks. On examination muscle tone in the legs was increased, while otherwise decreased. First cerebral MRI was performed at age 14 months and showed a leukodystrophy affecting most of the supratentorial white matter with sparing of the periventricular and infratentorial white matter (Fig. 2A). Over the next 11 months she made a slow but continuous recovery. At age 16 months she was able to fixate and hold objects again but was still unable to sit unsupported. By age 24 months no remaining abnormalities were found on neurological examination. Consistent with the clinical findings, a drastic improvement in white matter abnormalities was seen on cerebral MRI performed at age 23 months. The patient is now 25-years-old and works as a kindergarten teacher. Her only residual deficit is a very mild gait abnormality. Only residual gliotic lesions affecting the corpus callosum and periventricular white matter were seen on cerebral MRI at age 20 years (Fig. 2C).

Patient III.5, female, was born after an uneventful pregnancy and developed normally until age 10 months when all abilities were rapidly lost during a febrile illness. Like patient III.2, she was very irritable, showed no social contact and refused to eat necessitating tube feeding for 4 months. Muscle tone in the legs was increased, while otherwise decreased. Cerebral MRI at age 11 months also showed a leukodystrophy with pronounced signal changes affecting the deep cerebral white matter and to a lesser extent the nucleus dentatus (Fig. 2D). Over the following 20 months a slow but continuous improvement in her condition was observed. At age 17 months she was able to sit unsupported again and speak single words, at 21 months she could walk short distances and speak 2-word sentences and at 30 months her only reported deficit was an inability to run fast. In contrast to the positive clinical development, MRI imaging initially showed a further progression of white matter abnormalities until age 21 months (Fig. 2E) before gradually almost completely resolving. Between ages 8 and 10 years four focal seizures were reported. Now at age 24 years she works as a social worker, has a slight gait abnormality and avoids walking long distances. Only residual white matter changes remain on cerebral MRI (Fig. 2G).

Patient IV.1, female, daughter of patient III.5, was born after an uneventful pregnancy at term. After an initial normal period of development, her ability to sit, crawl and walk with support was lost within 2 days coinciding with a viral infection at age 12 months. She was very irritable, lying with extended, crossed legs. As in the two previous cases, the acute episode was followed by a gradual recovery of lost abilities. At 18 months she was able to sit unsupported again, crawl slowly and stand with support. When last seen at age 21 months she was walking short distances unsupported but speaking only a few single words. Cerebral MRIs obtained over a follow-up period of 16 months displayed a pattern of white matter abnormalities that resemble those seen in patients III.2 and III.5 (Fig. 2H–K). T2 hyperintensities were most pronounced in the centrum semiovale with a central strand of more normal appearing white matter. Interestingly, the apparent diffusion coefficient of the initial MRI study performed shortly after onset of symptoms revealed widespread distinctly restricted diffusion in the T2 lesional areas (Fig. 2L). In follow up studies, the apparent diffusion coefficient signal then reversed to bright intensities indicating enhanced diffusion (Fig. 2M–O).

None of the patients displayed any dysmorphic features and somatic growth including head circumference was normal. In all three patients, an extensive diagnostic work-up was performed. Analysis of plasma lactate in the acute stage was normal in patient III.2 but elevated in patient III.5 (3.8 mmol/l) and patient IV.1 (3.5 mmol/l) (reference 0.5–2.2 mmol/l). Normal values were seen for lactate in CSF (III.2, III.5, IV.1), CSF Protein (III.2, III.5, IV.1), copper, ceruloplasmin (III.2, III.5), carbohydrate-deficient transferrin (III.5, IV.1), carnitine and acylcarnitine (III.5, IV.1), very long chain fatty acids (III.2, III.5, IV.1), enzyme activities of arylsulfatase A, beta-galactosidase, hexosaminidase A, hexosaminidase B, galactosylceramidase (III.2, III.5, IV.1), amino acids in CSF and plasma (III.5, IV.1) and organic acids in urine (III.5).

Biopsy of skin and muscle from patient III.5 was performed. Activities of pyruvate dehydrogenase complex and respiratory chain enzymes in fibroblasts showed moderately reduced activities of complex III and complex IV in fibroblasts and reduced activities of complex II, III and IV in muscle. Muscle histology showed no abnormalities, in particular no ragged red fibres.

Nerve conduction velocity (III.2, III.5, IV.1) as well as acoustic evoked potentials (III.2, III.5, IV.1) were repeatedly normal. Visual evoked potentials were normal in III.2 and abnormal in III.5 and IV.1 in the acute phase.

### Proton MR-spectroscopy and Myelin sensitive imaging indicate mitochondrial failure and demyelination during the acute phase and subsequent recovery

**Figure 3:**
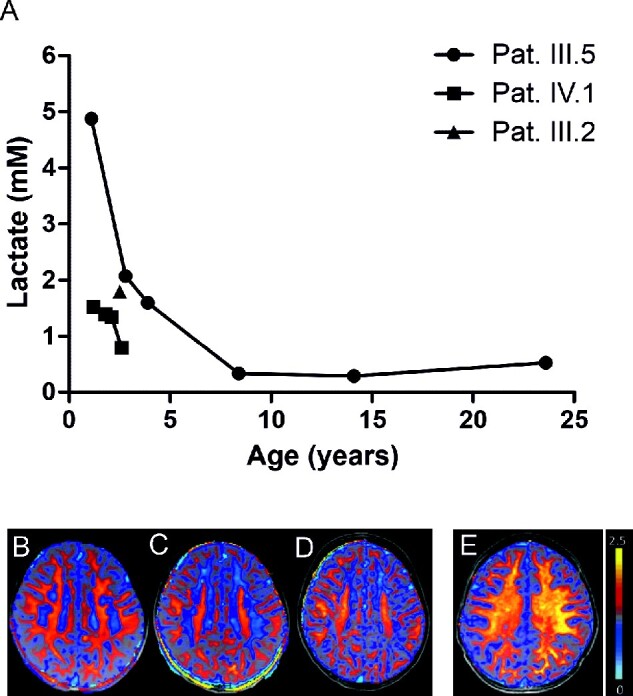
**Quantitative MR spectroscopy and Myelin sensitive imaging.** (**A**) Lactate levels (in mM) in patients III.2, III.5 and IV.1 at different time points. Over time lactate returned to normal ranges (≤ 1 mM) in the two follow up studies. (**B–D**) Overlay of colour-coded axial MTsat maps onto corresponding T1 weighted images of patient IV.1. At age 13 months in **B** blue colour-coded white matter areas depict myelin deficit; at 19 months in **C** extension of blue-grey colour-coded WM regions and appearance of light blue-coded WM frontally representing very low MT values (close to CSF) reveal ongoing demyelination; at 29 months in **D** appearance of grey-red-orange-yellow colour-coded WM areas illustrate remyelination. (**E**) Overlay of colour-coded axial MTsat map onto corresponding T1 weighted image of a healthy control age 18 m and the colour scale encoding the MTsat values. Note the predominance of red-orange coded MTsat values and transition to yellow coded WM pointing to sufficient myelination.

Localized MRS performed in Patients III.2, III.5 and IV.1 shortly after onset of symptoms uniformly revealed lactate elevation in white matter lesions in all three patients. In patient III.5 and her daughter, patient IV.1 follow up MRS were obtained over a period of 14 and 1.3 years, respectively. Results demonstrated a gradual normalization coinciding with the clinical improvement (Fig. 3A).

MTsat maps obtained in IV.1 depicted initial myelin deficit most pronounced in the lesions [blue colour-coded white matter (WM) areas, Fig. 3B–D]. In comparison to the maps of a younger healthy control (age 1.5 years) in Fig. 3E the myelin loss appeared more widespread than depicted by conventional T2 weighted imaging (Fig. 2H–K). Subsequent studies 6 months and 1.3 years later indicated progressive demyelination (Fig. 3C) followed by remyelination (appearance of grey-red-orange-yellow colour-coded WM, Fig. 3D).

### Exome sequencing detects a variant in FBP2 with unknown pathogenicity

Because segregation within the family was compatible with maternal inheritance, the complete mitochondrial genome from patient III.5 was sequenced. No variants with known or suspected pathogenicity or large-scale mitochondrial DNA rearrangement were detected. Furthermore, no deletions or duplications of mitochondrial DNA were seen.

Exome sequencing was initially performed in DNA samples from family members, III.2, III.5 and IV.1 and later extended to II.2, II.4, III.4 (Supplementary Table 1). No mutations in known leukodystrophy genes were detected but the analysis revealed heterozygous missense variants common in all patients in *CCT6A* (c.446T>C, p. Ile149Thr) and *FBP2* (c.343G>A, p. Val115Met) (Supplementary Table 2). The variant in *CCT6A* is a frequent multiallelic site in gnomAD and is predicted to have a benign impact (Supplementary Table 2). Therefore, it was not considered pathogenic. The variant p. Val115Met in *FBP2* is not described in our in-house population database and only once in a European male in gnomAD (accessed in May 2020) (Allele frequency: 0.000003979). The *FBP2* variant p. Val115Met is predicted to be possibly damaging by 10 out of 13 *in silico* analysis tools (Supplementary Table 3). Furthermore, the position Val115 is highly conserved both in vertebrate FBP2 and FBP1, and also in FBPs of insects and plants, as can be derived from a multiple sequence alignment and is highly conserved in terrestrial vertebrates (Supplementary Fig. 2A). Val115 is situated in a hydrophobic pocket formed by the residues Val94, Tyr140 and Ala153. Active site and metal binding sites are not directly involved but an exchange of the small valine side chain by the much larger methionine may disturb the structure locally (Supplementary Fig. 2B). Sanger sequencing was performed in all family members who agreed to the analysis (Supplementary Fig. 1). The *FBP2* variant was confirmed in the family members who had undergone exome sequencing and was also confirmed in patient IV.2 who has subsequently been shown to have leukoencephalopathy, developmental delay and behavioural problems. It was also detected in patient III.3 who has a history of psychiatric problem but declined MR imaging (Supplementary Fig. 1). The *FBP2* variant was not detected in the only healthy offspring, III.1. As the genetic results suggest a causative role of the *FBP2* c.343G>A, p. Val115Met variant in the disorder seen in the family we conducted biochemical analyses to support its pathogenicity.

### The V115M-FBP2 variant affects activity, substrate affinity and thermal stability of FBP2

**Figure 4:**
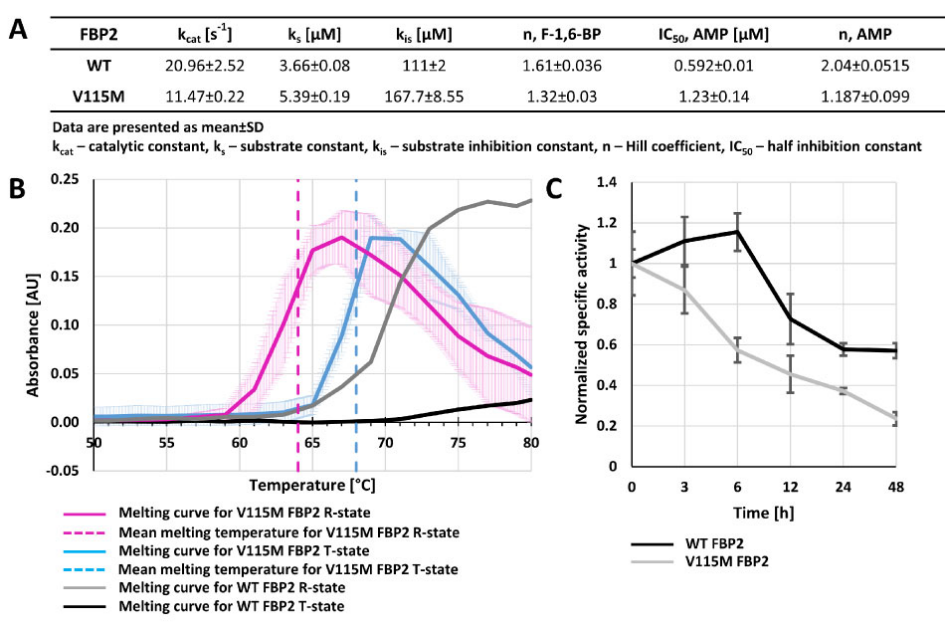
**Comparison of kinetics and stability of the FBP2 variants.** Comparison of V115M-FBP2 and WT-FBP2 kinetic parameters in **A**, and thermal stability of the mutant protein in **B**. In **B**, solid lines represent mean melting curves from three independent measurements, and dashed lines indicate mean melting temperatures. (**C**) Enzymatic activity of V115M-FBP2 and WT-FBP2 after prolonged incubation at 37°C. The activity was normalized to mean activity at *t* = 0 for the respective FBP2 form.

The residue 115 is not directly involved in the mechanism of catalysis and allosteric inhibition of FBP2. However, the analysis of kinetic properties of the recombinant protein demonstrated that V115M-FBP2 was about 2-fold less active than WT-FBP2 (Fig. 4A), and its affinity to the substrate, F-1,6-BP, was over 1.5 times lower. V115M-FBP2 was also significantly less sensitive to the action of the allosteric inhibitor AMP and the cooperativity of the inhibition was lost. Since the cooperativity of the allosteric inhibition of FBP2 is linked to the mechanism of R-to-T transition and tetramerization, the V115M mutation most likely disrupts the quaternary structure of the protein. Comparison of melting points of V115M-FBP2 and WT-FBP2 using the thermal denaturation method showed that, as in the case of WT-FBP2, the presence of AMP during incubation improved the thermal stability of V115M-FBP2 (Fig. 4B). However, both the AMP-saturated and the AMP-free V115M-FBP2 was significantly less stable than WT-FBP2. To verify these differences, we monitored activities of both enzyme variants during incubation at 37°C. Their activities decreased about 2-fold after the first 20–25 h of incubation, however, during the subsequent 25 h, activity of WT-FBP2 remained unchanged while activity of V115M-FBP2 was constantly decreasing (Fig. 4C).

### The V115M-FBP2 variant alters subcellular localization of FBP2

**Figure 5:**
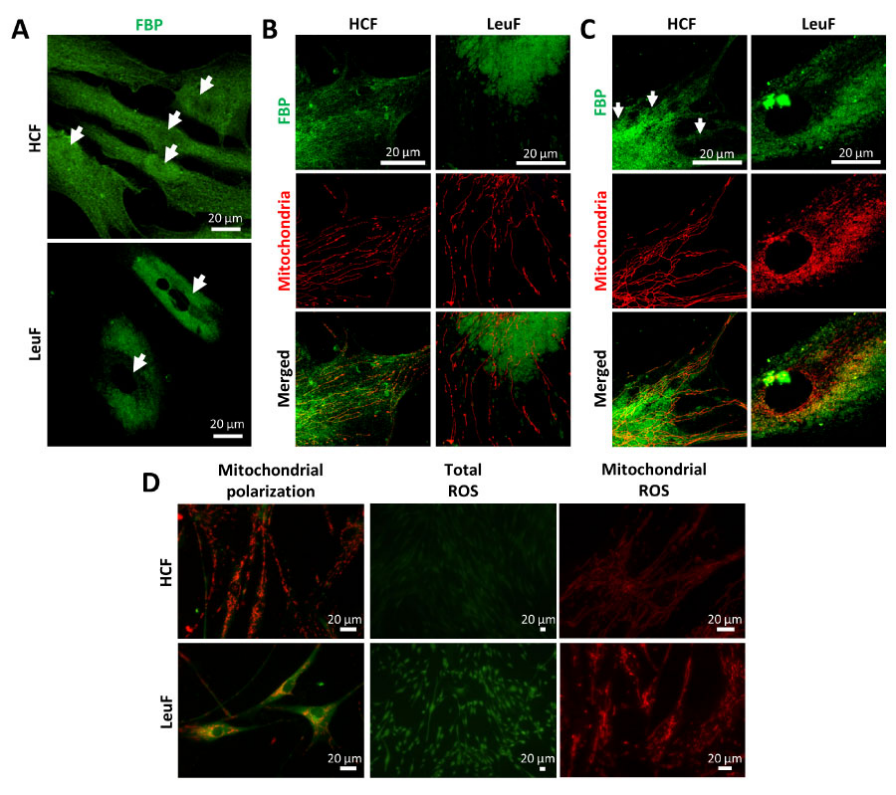
**Subcellular localization of the FBP2 variants.** (**A**) In healthy control fibroblasts (HCF), FBP2 is homogeneously distributed in whole cytoplasm and nuclei (arrows), as determined by immunocytochemistry. In fibroblasts derived from patient III.5 (Leukodystrophy Fibroblasts, LeuF), nuclei (arrows) are empty and the enzyme accumulates only in their vicinity, not in cellular protrusions. (**B**) GSK3β inhibition results in accumulation of FBP2-related immunofluorescent signal with mitochondria in HCF, but not in LeuF. (**C**) In control conditions, FITC-labelled V115M-FBP2 does not co-localize with mitochondria of HCF, while WT-FBP2 does (arrows). (**D**) Mitochondrial network in LeuF is much less developed and membrane of the organelles is less polarized than in HCF. Total as well as mitochondrial production of reactive oxygen species (ROS) in LeuF is higher than in HCF.

Next, we analysed the subcellular localization of FBP2 in healthy fibroblasts, and fibroblasts derived from patient III.5. As expected, in healthy control fibroblasts (HCF), FBP2 was localized in cytoplasm where it was relatively homogeneously dispersed in the whole cell. A slightly fibrillar localization of the enzyme might be attributed to colocalization of a fraction of the protein with mitochondria.^8^ The enzyme was also present in nuclei (Fig. 5A). In contrast, in fibroblasts derived from patient III.5, FBP2 showed granular localization. It was present only within the cells’ bodies, not in protrusions and did not localize in cells’ nuclei.

### The V115M-FBP2 variant does not colocalize with mitochondria, correlating with disturbance of their network and increase in ROS production

The localization of FBP2 in cytoplasm seen in fibroblasts from patient III.5, suggested mitochondrial accumulation of the enzyme, but immunostaining revealed that in fact, it did not co-localize with mitochondria, even if the cells were treated with GSK3β inhibitor (Fig. 5B and Supplementary Fig. 3) or elevated Ca^2+^ concentrations (data not shown) which have been shown to stimulate accumulation of FBP2 on mitochondria.^8^ Both factors increased colocalization of the FBP2-related fluorescence with mitochondria in HCF. To conclusively demonstrate that the lack of FBP2 colocalization with mitochondria is a result of inability of V115M-FBP2 to interact with the organelles we transfected HCF with FITC-labelled V115M-FBP2 and found no accumulation of the FITC-related fluorescence on mitochondria (Fig. 5C).

Next, we studied the mitochondrial network in fibroblasts from patient III.5. We found that the network was less developed and mitochondrial membranes were less polarized compared to HCF (Fig. 5D), as determined by about 2-fold reduction of the red-to-green fluorescence intensity ratio (from ∼2.8, SD = 1.1, *n* = 59 to ∼1.34, SD = 0.4, *n* = 57; *P* < 0.00001). Moreover, the disturbances in the mitochondrial network were accompanied by an increased production of ROS, both in cytoplasm (almost 2-fold increase, *P* < 0.00001) and in mitochondria (about 1.4x increase, *P* < 0.00001) (Fig. 5D and Supplementary Fig. 3).

### The V115M-FBP2 variant is degraded by ERAD and ER-to-lysosomes-associated degradation (ERLAD) pathways

**Figure 6:**
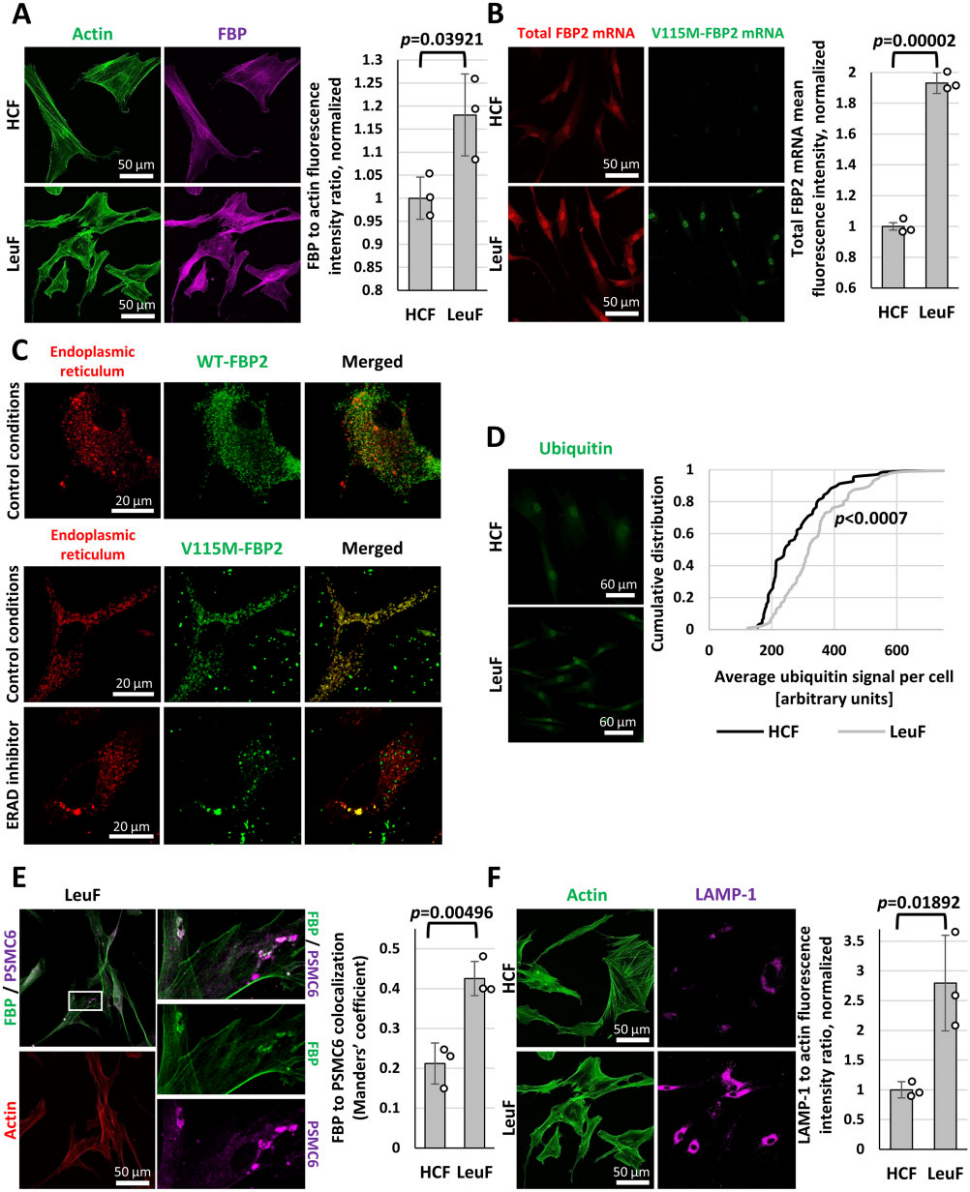
**Expression and degradation of the FBP2 variants.** (**A**) Total fluorescent signal associated with polyclonal antibodies against FBP is stronger in Leukodystrophy Fibroblasts (LeuF) than in healthy control fibroblasts (HCF). (**B**) Similarly, the signal for total FBP2 mRNA is much stronger in LeuF than in HCF. V115M-FBP2 mRNA is not detected in HCF. (C) In HCF, FITC-labelled WT-FBP2 hardly co-localizes with ER while V115M-FBP2 highly co-localizes with ER in control conditions, but not after ERAD inhibition. (**D**) Total fluorescent signal associated with K48 polyubiquitination is slightly but significantly stronger in LeuF than in HCF. (**E**) The majority of FBP-related granules co-localize with proteasome subunit PSMC6. (**F**) LAMP-1 level is over 2.5-times higher in LeuF than in HCF.

Surprisingly, the total fluorescent signal associated with polyclonal antibodies against FBP2 was much stronger in fibroblasts from patient III.5 than in HCF (Fig. 6A). This raised the question, if the elevation of FBP2-related signal was a result of increased expression of V115M-FBP2 and/or WT-FBP2. Since antibodies against FBP2 do not discriminate between these two variants we used fluorescently labelled oligonucleotides against both forms of FBP2 to quantify their expression. As expected, expression of V115M-FBP was only seen in fibroblasts from patient III.5, but not in HCF, demonstrating specificity of the oligonucleotides (Fig. 6B). Moreover, in fibroblasts from patient III.5, the expression of WT-FBP2 was increased. This prompted us to analyse the colocalization of WT-FBP2 and V115M, with various systems of protein turnover in a cell. We found that transfection of wild type fibroblasts with FITC-labelled V115M-FBP2 and WT-FBP2 led to colocalization of the mutant protein, but not the WT-FBP2, with the endoplasmic reticulum (ER) (Fig. 6C and Supplementary Fig. 3) ER is a part of the cellular machinery of misfolded protein degradation. Endoplasmic-reticulum-associated protein degradation (ERAD) is a process in which proteins are dislocated from ER to the cytosol for ubiquitylation and proteasomal degradation. Treatment of fibroblasts with the ERAD inhibitor Eeyarestatin decreased the level of FITC-labelled V115M-FBP2 colocalization with ER (Fig. 6C and Supplementary Fig. 3). In line with this, we observed a higher level of FBP2 (the sum WT-FBP2 and V115M-FBP2) polyubiquitylation in fibroblasts from patient III.5 compared to HCF (Fig. 6D). We also found that most of the V115M-FBP2-fluorescence colocalized with PSMC6 protein which is a component of the 26S proteasome, although the total level of PSMC6 in these cells was not elevated (Fig. 6E). It is known that some defective proteins do not engage ERAD machinery because their aggregates are too large and/or cannot be relocated across the ER membrane (ERAD-resistant misfolded proteins).^26^ The low thermal stability of the V115M-FBP2 and the presence of the enzyme in granule-like structures, suggested that the protein may be only partially eliminated by ERAD. Instead, it may be predominantly cleared by the alternative, ERAD-independent process of misfolded proteins degradation referred to as ELRAD (ER-to-lysosome-associated degradation), involving ER-phagy, microautophagy or vesicular transport.^27^ One of the indicators of the ELRAD process intensity is the abundance of lysosome-associated membrane glycoprotein 1 (LAMP-1) which represents the major constituent of lysosomal membrane proteins in a cell.^27^ Results of our study showed that the expression of LAMP-1 protein was over 2.5-times higher in patient III.5 fibroblasts than in HCF (Fig. 6F). This points to ELRAD as the main system involved in the clearance of defective variant of FBP2.

## Discussion

In this article, we describe a family with a novel white matter disease. The three index patients experienced a severe crisis during a febrile illness at age 10–13 months resulting in a loss of all learned abilities. The acute episode was followed by a gradual recovery phase and achievement of normal developmental status within 1–2 years. Little is known about the early childhood of patients II.1 and II.2, as both grew up in an orphanage because their mother was unable to care for them due to a psychiatric disorder. However, of note is that they both display a similar residual MRI pattern to their daughters. A further family member, patient III.4, also has documented reversible white matter changes on repeated MRI imaging. Remarkably, none of the index patients has experienced a second attack indicating that there might be a phase of vulnerability in brain development around the end of the first year of life.

Cerebral MRI at the time of the crisis revealed dramatic leukodystrophy of the periventricular white matter and the corpus callosum, more severe than in any of the known remitting leukodystrophies. White matter changes in the T2 weighted images were seen to increase for several months despite clinical improvement. In patient IV.1 diffusion-weighted magnetic resonance imaging, myelin imaging and MRS were performed. Diffusion-weighted magnetic resonance imaging in the initial acute phase showed restricted diffusion in the affected white matter indicating cytotoxic oedema consistent with a metabolic crisis. This finding was supported by MRS evidence of increased lactate in the white matter. During the following months diffusion was temporarily increased compatible with a reduction of myelin and subsequent remyelination. Consistently, myelin imaging initially demonstrated a massive decrease followed by a gradual increase and almost normalization of white matter myelin. The imaging data together with the clinical course are best compatible with an event associated with massive demyelination followed by remyelination over a period of 1–2 years. The near complete recovery makes preferential neuronal damage unlikely. We therefore hypothesize that the primary affected cells in this disorder are the oligodendrocytes.

Following exclusion of mutations in the mitochondrial DNA and in known leukodystrophy genes, exome sequencing resulted in the detection of only one variant that segregated with the leukodystrophy in the family and not frequently reported in current databases; *FBP2* c.343G>A, p. Val115Met. The affected amino acid Val115 is highly conserved in terrestrial vertebrates and is predicted to be damaging. In all available family members, the variant segregated with leukoencephalopathy on Sanger sequencing of the *FBP2* gene. Subject III.1 is the only offspring who carries the WT allele. She has no known neurological disorder and normal cranial MR imaging (data not shown).

So far, no human disorders have been linked to variants in the *FBP2* gene. Mutations in *FBP1*, coding for FBP1, a key enzyme of gluconeogenesis that is expressed in liver and kidney, cause fructose-1,6-bisphosphate deficiency, a disorder characterized by recurrent severe metabolic crises after fructose ingestion or during febrile illness. To assess the pathogenicity of the V115M-FBP2 variant, we investigated enzymatic function, in particular the kinetic properties and stability of the variant. We found that V115M-FBP2 has reduced activity, thermal stability and substrate affinity. Moreover, the enzyme was less sensitive to the action of allosteric inhibitor AMP and the cooperativity of the inhibition was lost. Under physiological conditions FBP2 forms dimers and tetramers, thus we speculate that WT-FBP2 and V115M-FBP2 co-expressed in a cell form heteromers with reduced enzyme activity and stability explaining a dominant negative effect of the variant. The role of gluconeogenesis in the brain, where FBP2 but not FBP1 is expressed, is not fully understood but there is evidence that it is an important part of the local energy metabolism under physiological and pathological conditions.^28^ In fructose-1,6-bisphosphate deficiency of the liver enzyme FBP1, acute attacks with hypoglycemia and lactic acidosis are often triggered by febrile infections. In the three index patients described here, the severe crisis also followed a febrile infection and lactate was elevated in the white matter during the acute phase on MRS. These findings indicate that defective gluconeogenesis might contribute to the pathophysiology. As synthesis of myelin is a very energy consuming process the oligodendrocytes might be particularly vulnerable to such a defect during the phase of intense myelination at the end of the first year of life.^29^

On fibroblast analysis, WT-FBP2 was increased in the fibroblasts of patient III.5. Presumably, this was due to reduced FBP2 function in the cell, and subsequent upregulation of FBP2 expression. We were also able to demonstrate preferential clearing of the defective protein by ERLAD leading to upregulation of LAMP-1 expression. These findings further support pathogenicity of the variant.

As recently reviewed by Huangyang et al.,^30^ insights from cancer research have shown that many metabolic enzymes have activities outside of their established metabolic roles, including the regulation of gene expression, DNA damage repair, cell cycle progression and apoptosis. The authors speculate that modulating gene expression by metabolic enzymes facilitates adaptation of the cell to rapidly changing environments. A recently described example of a genetic defect that only affects the non-canonical functions of a metabolic enzyme involves the HK1 gene. Autosomal recessive mutations in the HK1 gene, coding for hexokinase 1, lead to a loss of enzymatic function and cause non-spherocytic haemolytic anaemia. However, heterozygous de novo missense mutations in the same gene detected recently in six patients with developmental delay, intellectual disability, structural brain abnormality and visual impairment, were associated with normal hexokinase activity.^31^

FBP2 reportedly also has several other functions in addition to enzymatic activity. Firstly, an involvement in mitochondrial protection has been shown. Upon activation of glycolysis by insulin, FBP2 colocalizes with mitochondria resulting in a reduced rate of calcium-induced mitochondrial swelling and protection of ATP synthesis.^8^ We analysed the effect of the V115M-FBP2 variant on mitochondria in fibroblasts of patient III.5 and found a pronounced disturbance of the mitochondrial network. In fact, the V115M-FBP2 variant did not colocalize with mitochondria, and ROS production in the organelles and whole cell was increased. Also supportive of mitochondrial dysfunction was evidence of elevated lactate levels in the white matter during the acute attack and subsequent normalization over the following months coinciding with clinical improvement. Interestingly, the MRI pattern seen in our patients matches that seen in Kearns–Sayre or Leigh syndrome, both classical mitochondrial disorders.^32^ However, such patients have a progressive disease course with grey matter pathology especially of the basal ganglia which was not seen in the patients described here. In conclusion, while we collectively have evidence suggesting the pathophysiology of the disorder described in this article involves mitochondrial dysfunction, it does not, however, resemble a classical mitochondrial disorder.

A further function attributed to FBP2 is regulation of nuclear processes. Like many glucose metabolism enzymes, FBP2 has a classical Nuclear Localization Sequence.^33^ FBP2 interacts with histone and non-histone nuclear proteins and has been found to restrain mitochondrial biogenesis and respiration by colocalization with the c-Myc transcriptions factor.^34^^,^^35^ Furthermore, in cardiomyocytes and cancer cells, FBP2 has a recognized role in the regulation of the cell cycle.^36^ Immunostaining patient III.5 fibroblasts we were not able to detect FBP2 in nuclei. This suggests a dominant negative effect of V115M-FBP2 on nuclear localization, presumably due to heteromerization of WT and mutant proteins and indicates that the nuclear functions of FBP2 are diminished in *FBP2* related-leukodystrophy.

We conducted a search using the GeneMatcher database^37^ for further families with a similar phenotype and variants in the *FBP2* gene and inquired with other international specialists in leukodystrophies if they had seen similar cases but were so far unsuccessful. It is possible that only variants that affect both the enzymatic function and the non-canonical functions of FBP2 will be associated with the clinical phenotype seen in the family described here. Furthermore, FBP2-related leukodystrophy is, no doubt, an extremely rare disorder and misinterpretation as an autoinflammatory disorder such as acute disseminated encephalomyelitis, the initial diagnosis in patients III.2 and III.5, may hamper recognition of further cases.

In conclusion, we report on a novel disorder that we termed *FBP2*-related leukodystrophy. The phenotype is characterized by a metabolic crisis at the end of the first year of life leading to widespread demyelination and loss of abilities. The patients recover over several months regaining normal development accompanied by remyelination. The variant in *FBP2* that was found to segregate in the family with the disorder, damages not only the enzymatic activity but also the non-canonical functions of FBP2 involved in mitochondrial protection and nuclear localization. FBP2-related leukodystrophy is a novel member of the group of remitting leukodystrophies.

## Supplementary material

Supplementary material is available at *Brain Communications* online.

## Funding

This work was supported by the German Research Foundation (DFG, Ga354/14–1, HU 941/2–5), die Niedersächsisches Ministerium für Wissenschaft und Kultur [11–76251-12–4/12 (ZN2938)] and by the Polish National Science Centre (UMO-2015/19/B/NZ1/00332).

## Competing interests

The authors report no competing interests.

## Supplementary Material

fcab036_Supplementary_Data

## Abbreviations

ADC =: apparent diffusion coefficient
AMP =: adenosine monophosphate
BSA =: bovine serum albumin
DWI =: diffusion-weighted magnetic resonance imaging
EDTA =: ethylenediaminetetraacetic acid
ER =: endoplasmic reticulum
ERAD =: endoplasmic reticulum-associated protein degradation
ERLAD =: ER-to-lysosomes-associated degradation
F-1,6-BP =: fructose-1,6-bisphosphate
FBP1 =: fructose 1,6-bisphoshatase
FBP2 =: muscle fructose 1,6-bisphosphatase
FITC =: fluorescein isothiocyanate
FLAIR =: fluid-attenuated inversion recovery
HCF =: healthy control fibroblasts
Het =: heterozygous mutated
LAMP-1 =: lysosome-associated membrane glycoprotein 1
LeuF =: leukodystrophy Fibroblasts
mRNA =: messenger RNA
MRS =: magnetic resonance spectroscopy
MT =: magnetization transfer
mtDNA =: mitochondrial DNA
MTsat =: MT saturation
NAD+ =
NAD+ =: nicotinamide adenine dinucleotide
=: nicotinamide adenine dinucleotide phosphate
PBS =: phosphate-buffered saline
ROS =: reactive oxygen species
WES =: whole exome sequencing
WM =: white matter
WT =: wild type
