# Supplementary material for: A novel remitting leukodystrophy associated with a variant in FBP2
Source: Brain Commun. 2021 Mar 11;3(2):fcab036. doi: 10.1093/braincomms/fcab036 (PMC8097510; doi:10.1093/braincomms/fcab036)
Supplement: fcab036_Supplementary_Data [file fcab036_supplementary_data.zip › Supplementary material.pdf]

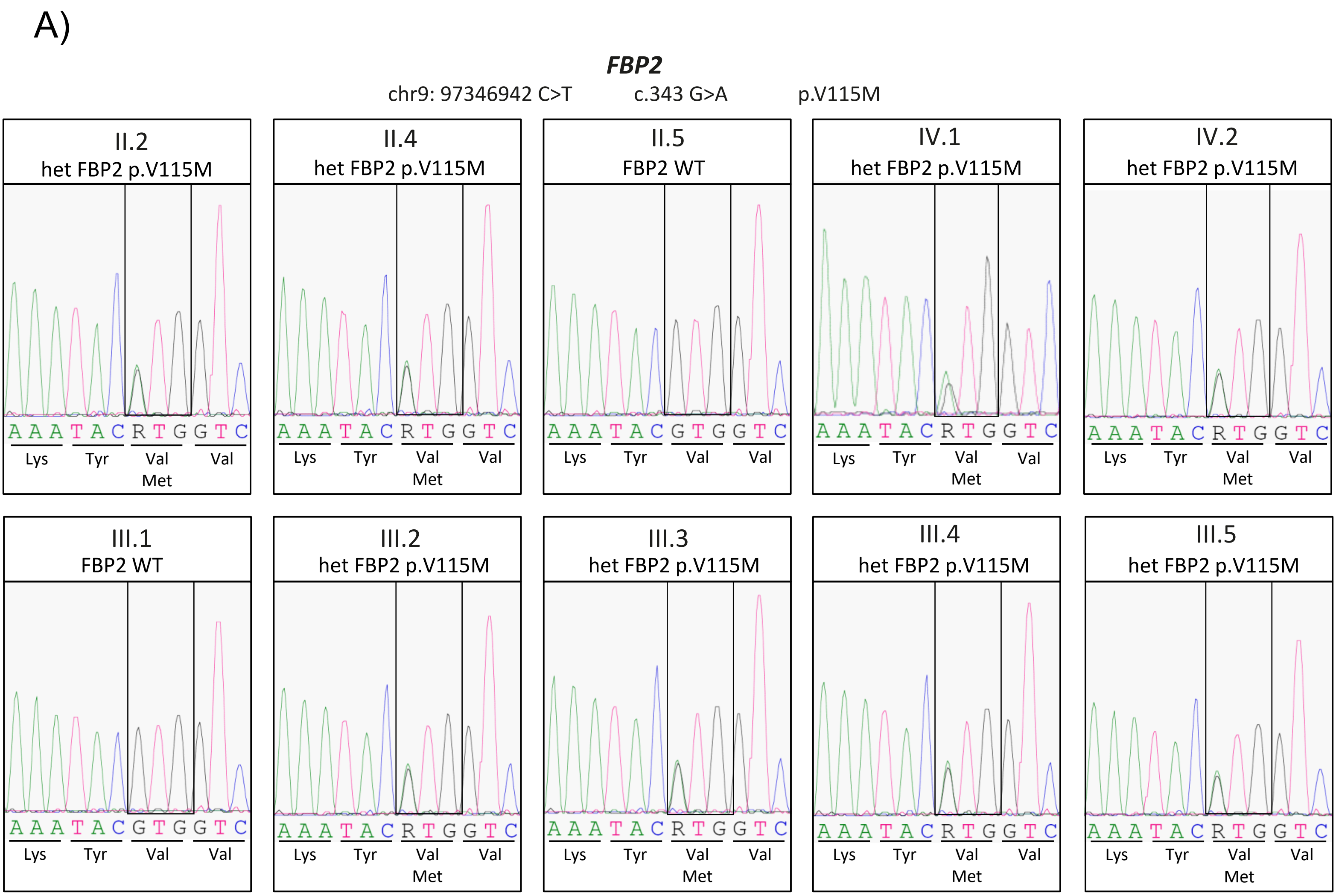

Supplementary Figure 1: Confirmation of heterozygous FBP2 variant. A) Sanger sequencing chromatogram of the FBP2 mutation side is shown for each investigated family member with different mutation status wild type (WT) and heterozygous mutated (het). The position of the affected FBP2 nucleotide triplet coding for p.V115M is highlighted by a box. Nomenclature is according to GenBank accession number NM_003837.2 and NP_003828.2.

A)

```
                                130       140       150       160       170       180
                                  |         |         |         |         |         |
                                                                               *V115M
F16P2_HUMAN             IAGSVNVTGDEVKKLDVLSNSLVINMVQSSYSTCVLVSEENKDAIITAKEKRGKYVVCFD
Bos_taurus              IAGSVNVTGDEVKKLDVLSNALVINMLQSSYSTCVLVSEENKEAIITSKEKRGKYVVCFD
Gallus_gallus_FBP2      IAGTVNVTGDEVKKLDVLSNSLVINMLQSSYSTCVLVTEENKEALITPKEKRGKYVVCFD
Xenopus_tropicalis      ISGSVNVTGDEVKKLDVLSNDLVINMLKSSYGTCALVSEENKEVIIIPKEMQGKYVVCFD
Danio_rerio             IAGQVNVTGDEQKKLDVLSNDLIINLLQASYGTCLMVSEENKDAIYTPAEKRGKYIVCFD
human_FBP1              IAGSTNVTGDQVKKLDVLSNDLVMNMLKSSFATCVLVSEEDKHAIIVEPEKRGKYVVCFD
Anopheles_Gambiae_FBP   ISGDTNVQGEQVKKLDVLSNEIFINMLKSSYATCLLVSEENDNVIEIETDKRGKYVVSFD
Glycine_max_FBP1        VQGAVNVQGEDQKKLDVVSNEVFSNCLRSSGRTGIIASEEEDVPVAVEESYSGNYIVVFD
Aspargillus_niger_FBP   LAGSSNTTGDDQKKLDVIGNDIFISAMKGSGKCRILVSEEEEEAIVFDEHPNARYAVVCD
                        : *  *. *:: *****:.* :. . ::.*     :.:**:.  :       ..* *  *
Prim.cons.              IAGSVNVTGDEVKKLDVLSNDLVINML2SSYSTCVLVSEENKEAIIT2KEKRGKYVVCFD

                                190       200       210       220       230       240
                                  |         |         |         |         |         |
F16P2_HUMAN             PLDGSSNIDCLASIGTIFAIYRKTSE--------------DEPSEKDALQCGRNIVAAGY
Bos_taurus              PLDGSSNIDCLASIGTIFAIYRKTSE--------------DEPSEKDALQPGRNIVAAGY
Gallus_gallus_FBP2      PLDGSSNIDCLAPIGTIFAIYKKETD--------------DEPSEKDALQPGRKIVAAGY
Xenopus_tropicalis      PLDGSSNIDCLASIGTIFAIYRKTTD--------------TEPCEQDALQPGRNIVAAGY
Danio_rerio             PLDGSSNIDCLAPIGTIFAIYKRISD--------------GEPSEKDALQPGNQIVCAGY
human_FBP1              PLDGSSNIDCLVSVGTIFGIYRKKST--------------DEPSEKDALQPGRNLVAAGY
Anopheles_Gambiae_FBP   PLDGSSNIDCLVSIGSIFAITKQVKEN-------------TTPSEQDALQPGNKIVAAGY
Glycine_max_FBP1        PLDGSSNIDAAVSTGSIFGIYSPNDECLADIDDDPTLDTTEQRCIVNVCQPGSNLLAAGY
Aspargillus_niger_FBP   PIDGSSNLDAGVSVGTIFGIFKLPDEVLGPN---------NKVTAQDLLRAGTEMVASGF
                        *:*****:*. .. *:**.*                          :  : * :::.:*:
Prim.cons.              PLDGSSNIDCLASIGTIFAIY2KTSE3L222DDDPTLDTTDEPSEKDALQPGRNIVAAGY
```

B)

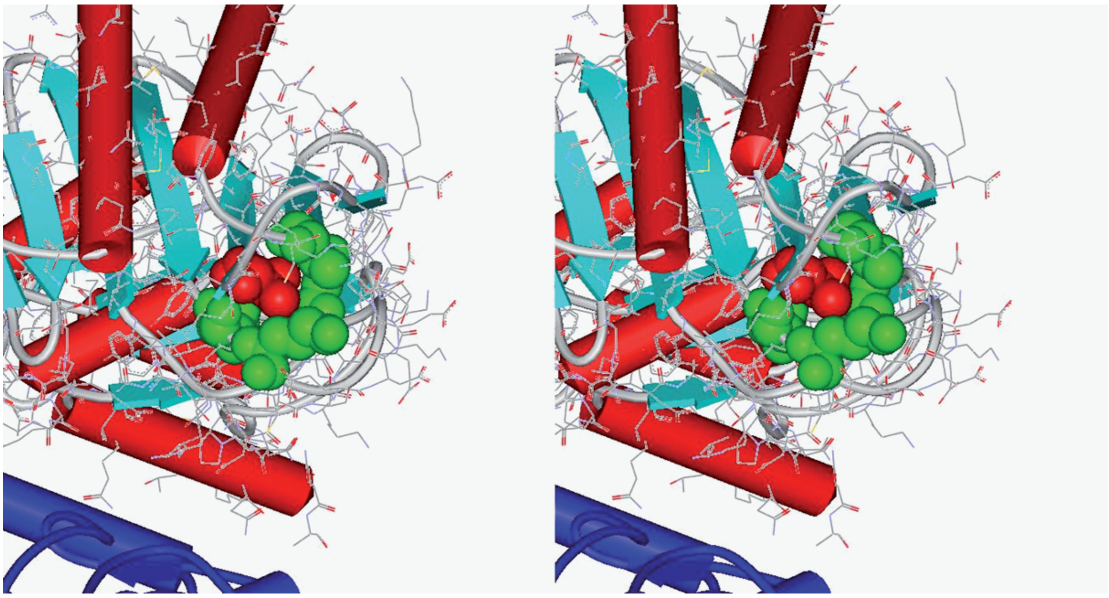

Supplementary Figure 2: Multiple Alignment and stereo representation of Val115 position in the FBP2 structure. A) The multiple alignment shows that the Val115 position is strongly conserved with hydrophobic residues for FBP in vertebrates, but also insects, plant, whereas there is an Ala as an even smaller hydrophobic side chain with Aspargillus. B) Stereo view „crossed eye“ of FBP2 subunit A with structural elements in schematic representation. Val115 space filling in red, surrounding hydrophobic residues Val94, Tyr140, Ala153, and the carbon backbone of Lys142 side chain in green, subunit C in dark blue. The figure was drawn with WebLab-Viewer (Molecular Simulations Inc., Burlington, MA, USA), using the X-ray structure of human FBP2 (pdb code 5et7).

Supplementary Figure 3. Comparison of reactive oxygen species (ROS) production and FBP2 variants colocalization with mitochondria and endoplasmic reticulum.

A) Quantification of reactive oxygen species ROS production. Both mitochondrial and total ROS production was increased in LeuF (leukodystrophy fibroblasts) as compared to HCF cells (healthy control fibroblasts); n – number of cells used for the fluorescence measurement.

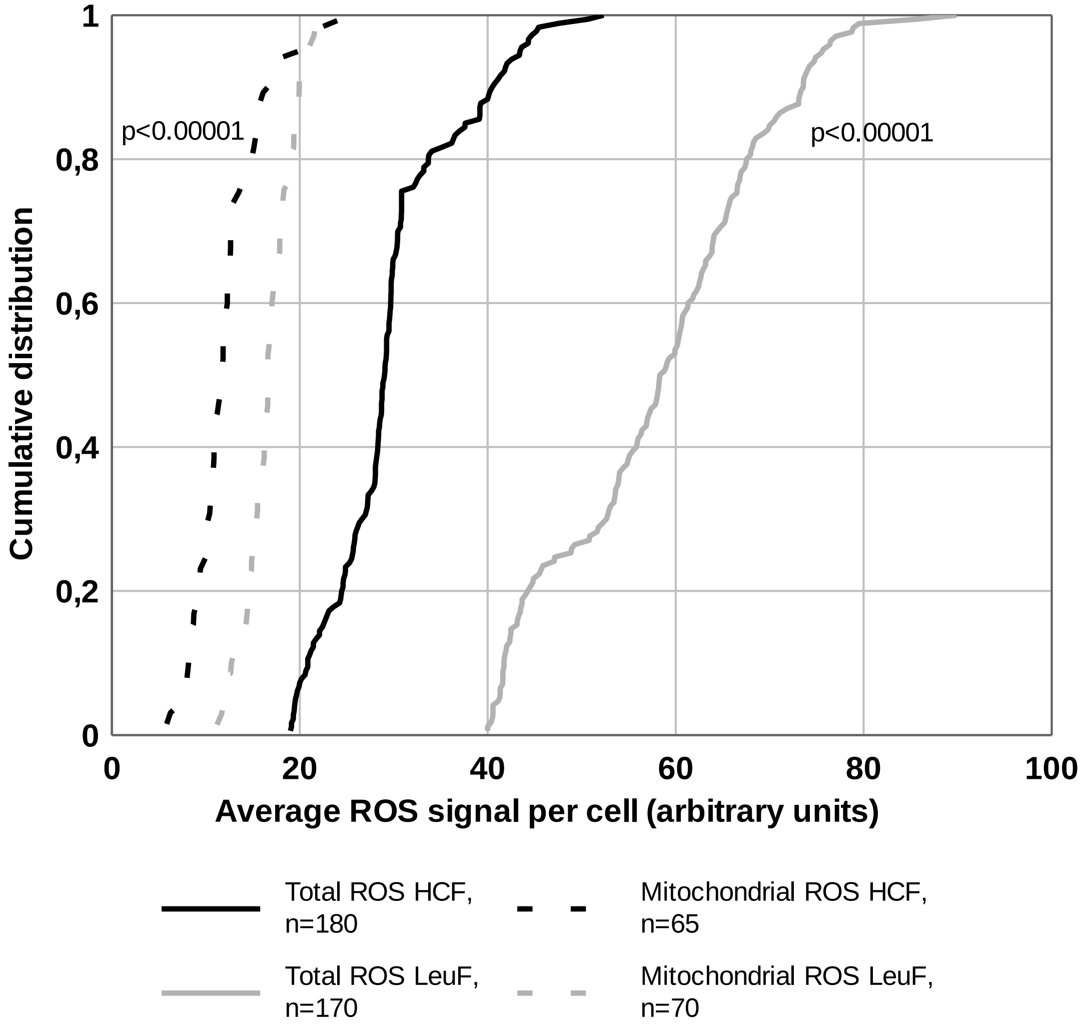

B) Colocalization of FBP2 variants with mitochondrial and endoplasmic reticulum networks in different contitions presented as Manders' coefficient which varies from 0 (no colocalization) to 1 (100% of colocalization). The coefficient was determined using the JACoP plugin of ImageJ[1]. The measurements were taken from at least 40 cells from at least 18 randomly selected areas. Data is presented as mean and standard deviation. * $p<0.05$, ** $p<0.0001$.

HCF - healthy control fibroblasts; LeuF - leukodystrophy fibroblasts; ctrl – control conditions; GSK3i – GSK3 inhibitor treatment; ERADi – treatment with endoplasmic reticulum (ER)-associated protein degradation inhibitor.

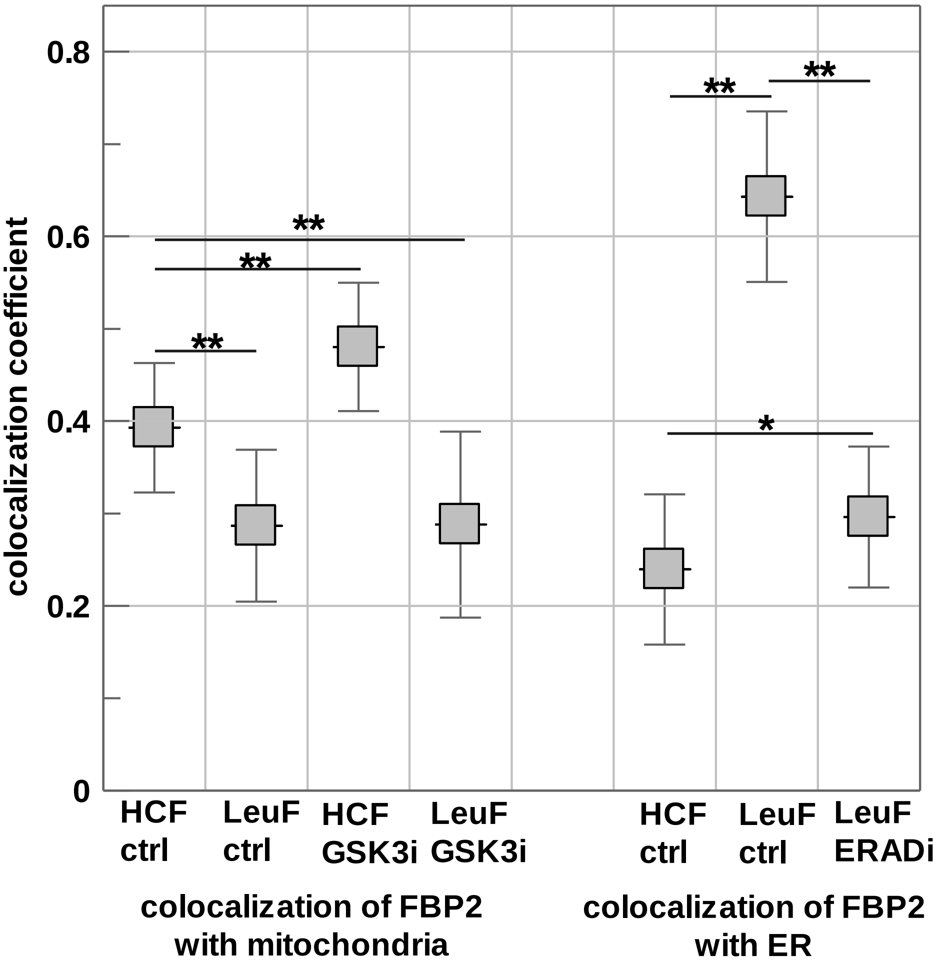

1. Bolte S, Cordelières FP, A guided tour into subcellular colocalization analysis in light microscopy. Journal of Microscopy, 2006;224: 213-232.

Sample	Varbank Pipeline	read length	Mean Cov	Cov 30x	Total reads	Unique mapped reads [%]	autosomal Runs of Homozygosity [Mb]	% of rare hom variants	Gender
II.2	v 3.5	101	95	86.0	122789264	79.00	15	0.1	Female
II.4	v 3.5	101	83	83.1	107980136	78.85	21	0.4	Female
III.2	v 3.4	76	75	81.9	132206850	74.67	8	0.1	Female
III.4	v 3.4	76	66	72.9	115661122	74.87	7	0.1	Female
III.5	v 3.5	101	74	76.9	100459852	82.61	34	0.0	Female
IV.1	v 3.5	101	73	80.7	106791788	87.73	14	0.0	Female

Supplementary Table 1: Basic whole exome sequencing (WES) information and output data. Overview of coverage, mapping information and homozygosity for each WES sample. Consanguinity can be estimated using % of rare hom variants (3 – 6%) and autosomal runs of homozygosity (> 200 – 300 mb). Cov, coverage; hom, homozygosity

								Sample					
**Gene**	**Chr**	**Position**	**Ref**	**Mut**	**Transcript ID**	**cDNA**	**Protein**	**II.2**	**II.4**	**III.2**	**III.4**	**III.5**	**IV.1**
CCT6A	7	56055733	T	C	NM_001762.3	c.446T>C	p.Ile149Thr	het	het	het	het	het	het
FBP2	9	94584660	C	T	NM_003837.3	c.343G>A	p.Val115Met	het	het	het	het	het	het

Supplementary Table 2: Detected filtered common variants. Detailed variant information with localization, substitution and occurrence within the family. Het, heterozygous

			CCT6A NM_001762.3		*FBP2* NM_003837.3	
	**Tool**	**Range**	**p.I149T**	**Prediction**	**p.V115M**	**Prediction**
	Median Rank Score	0 - 1	0.48		0.65	
FUNCTIONAL PREDICTION SCORES	SIFT	0 - 1	0.176	Tolerated	0.011	**Damaging**
	PolyPhen2	0 - 1	0.009	Benign	0.999	**Damaging**
	LRT	0 - 1	0.000290	Neutral	0.000	**Damaging**
	MutTaster	0 - 1	0.998	**Damaging**	1.000	**Damaging**
	MutAssessor	-5.545 - 5.975	1.3	Low functional impact	2.535	**Medium functional impact**
	FATHMM	-16.13 - 10.64	-1.15	Tolerated	-0.74	Tolerated
	PROVEAN	-14 - 14	-2.39	Neutral	-2.26	Neutral
	VEST3	0 - 1	0.195	Tolerated	0.638	**Damaging**
	MetaSVM	-2 - 3	-0.7441	Tolerated	0.1394	**Damaging**
	MetaLR	0 - 1	0.2964	Tolerated	0.5455	**Damaging**
	CADD	-7.5350 - 35.7885	1.3315	Neutral	5.6513	**Damaging**
	DANN	0 - 1	0.8208	Neutral	0.9987	**Damaging**
	FitCons	0 - 1	0.7354	**Functional important**	0.5731	Less Functional important
CONSERVATION SCORES	GERP	-12.3 – 6.17	2.39	**High conserved**	4.08	**High conserved**
	PhastCons	0 - 1	0.996	**High conserved**	0.980	**High conserved**
	PHYLOP20	-13.282 to 1.199	0.824	**High conserved**	0.876	**High conserved**

Supplementary Table 3: Detected filtered common variants. Detailed variant information corresponding bioinformatic prediction on the functional effect, conservation and occurrence in ExAC. Variant classification for each tool was obtained using dbNSFP version 3.4.
