# Supplementary material for: A novel remitting leukodystrophy associated with a variant in FBP2
Source: Brain Commun. 2021 Mar 11;3(2):fcab036. doi: 10.1093/braincomms/fcab036 (PMC8097510; doi:10.1093/braincomms/fcab036)
Supplement: fcab036_Supplementary_Data [file fcab036_supplementary_data.zip › Review_files.zip!/Original Submission - Response to Reviewers.pdf]

Original Submission – Response to Reviewers

Dear Dr. Bras,
Thank you very much for considering our article titled " A novel remitting leukodystrophy associated with a variant in FBP2" for publication in Brain Communications and thank you to the reviewers for their work and helpful advice. We have answered the concerns below point by point and changed the text accordingly.

Yours sincerely

Peter Huppke

Reviewer(s)' Comments to Author:

Reviewer: 1

Comments to the Author
The work of Gizak et al. shows for the first time the association of mutations in FBP2 with the development of an Autosomal Dominant Leukodystrophy, an association concluded by familial segregation with the disease, enzymatic function of the protein and analysis of patient fibroblasts. I believe the manuscript should be considered for publication with major revisions due to the lack of important quantifications in some results, nevertheless the amount of data provided is enough to conclude the association if quantification is provided. The fact that is a newly discovered association is also of major importance in the genetic diseases field.
Clinical description and genetic analysis are well characterized, and I would not recommend any changes, but experimental work should be improved mainly by providing quantifications to avoid any conclusion based on qualitative only analysis.
Major revisions:
Figure 5: I would suggest to please provide quantification of co-localization analysis when describing less or more co-localization in the results session. The total absence of co-localization of FBP2 with nuclei can be shown only qualitatively by co-staining with a nuclear marker. I suggest that co-localization with mitochondria should be properly evaluated. Mitochondrial polarization and ROS should also be quantified.
Figure 6: For the same reason, please provide co-localization analysis for Figure 6C and Figure 6E.

We have evaluated and quantified the parameters and described used procedures in Materials and Methods ("Immunocytochemistry" and "Measurements of cellular ROS production and mitochondrial polarization"). The obtained results are presented in the Results ("The V115M-FBP2 variant does not colocalize with mitochondria, correlating with disturbance of their network and increase in Reactive Oxygen Species (ROS) production") or as the Supplementary Figure 3.
We did not include the quantification of FBP2 nuclei because nuclei in LeuF fibroblasts are unequivocally empty (the analysis of several images shows that the intensity of the fluorescent signal from nuclei is in general equal to the background signal).

Minor comments: Please check word Co-localization or colocalization.

We changed co-localization into "colocalization" throughout the manuscript.

Reviewer: 2
Comments to the Author
The authors describe a family with a dominant form of early-onset remitting white matter disorder affecting eight members in four generations. Detailed clinical history and data including longitudinal MRI are reported for three patients. In these patients, the disease manifested around one year of age with sudden global disability following mild febrile illness. Neurological symptoms improved with almost complete recovery within two years. Neuroimaging showed marked demyelination that progressed for several months and was followed by remyelination. For other four patients, clinical history is not detailed and only MRI in adulthood is shown. Given the maternal transmission of the disease, mtDNA was investigated with no evidence of pathogenic sequence variants nor duplications/deletions. WES revealed the presence of a highly conserved missense variant p.Val115Met in the *FBP2* gene encoding muscle fructose 1,6-biphosphatase. The variant segregated with the disease being present in all affected members and absent in the only nonaffected member.
The authors performed several biochemical and cell biology experiments to support the pathogenic nature of the variant. In particular, they found that the variant 1) has a dominant negative effect on enzymatic activity and thermal stability of FBP2; 2) is associated with loss of FBP2 colocalization with mitochondria and nuclei, increased ROS production and abnormalities of the mitochondrial network.

Major comments:
1) the functional evidence for a pathogenic role of the *FBP2* variant is convincing. Biochemical and cell biology experiments are appropriately designed and performed
2) the study disclosed a prominent involvement of mitochondria as demonstrated by disturbances of mitochondrial network and ROS accumulation. Furthermore, plasma lactate was elevated in two patients and activities of respiratory chain complex II, III, and IV was reduced in muscle of one patient. Given the maternal transmission of the disease, the authors performed sequencing of the entire mtDNA and excluded the presence of mtDNA duplications and deletions. I believe that mtDNA studies should be completed by performing quantitative analysis in muscle in order to exclude mtDNA depletion.

mtDNA depletions had been excluded at the time but this was not mentioned in the article. We have included this in the methods and the results.

Minor comment:
On p. 19, 1st par, lines 1-4: to avoid ambiguity, reference for GeneMatcher (Sobreira *et al.*, 2015) should not be placed at the end of the sentence but where "GeneMatcher database" is mentioned (line 1)

The text was altered according to the reviewer’s suggestions.

Editorial Office comments:

-Please supply a graphical abstract. A graphical abstract is a visual representation of the central finding or methodology of the paper. It will be displayed in the table of contents. Authors should strive to make the graphical abstract informative, interesting, visually appealing, and straightforward (no caption needed, no abbreviations except gene names). Please upload as a separate high resolution image file (tiff preferred). The technical requirements for the graphical abstracts are:
--size of the submitted image: 1200 pixels square at 300dpi
--font of the text: Arial 12-16 point (smaller fonts will not be legible online)

A graphical abstract was provided

-Please add an Abbreviated Summary (up to 50 words) of your paper that captures the main purpose and conclusions of the work. The summary should state the main results written in the third person (‘[1st author surname] et al. report that …’) and conclusion. This summary will be used in the contents list online and should not contain any abbreviations other than those on the accepted abbreviations list and accepted gene/protein names, making it accessible to the non-specialist. Please upload it as a Word document. Allowed abbreviations: AIDS; ANOVA; AMPA; ATP; A,T,C,G; CNS; CSF; CT; DNA; ECG; EEG; EMG; GABA; HIV; MRI; NMDA; PET; PCR; RNA.

The abbreviated summary was added

-Please include a paragraph about data availability in the 'Materials and Methods' section. We strongly encourage data sharing either as supplementary information or uploads to repositories which you can link in the paper.

The paragraph was included

- To comply with our ethical standards, the appropriate checklist must be included (see Reporting Guideline section within 'Instructions to authors').

The way I understood the guidelines, not checklist is needed.

-Fig4b: please, replace green colour with magenta to make the figure color blind friendly and show single data points as n<10.

The figure was altered according to the reviewer’s suggestion.

-Fig6: please, provide how many times the experiment was repeated and how many cells were considered/experiment.

We have added the information to the Materials and Methods section or to the appropriate part of Results and Figure legends.
