# Supplementary material for: A novel remitting leukodystrophy associated with a variant in FBP2
Source: Brain Commun. 2021 Mar 11;3(2):fcab036. doi: 10.1093/braincomms/fcab036 (PMC8097510; doi:10.1093/braincomms/fcab036)
Supplement: fcab036_Supplementary_Data [file fcab036_supplementary_data.zip › Review_files.zip!/Original Submission-Decision Letter.pdf]

Original Submission – Decision Letter

22-Dec-2020

Dear Dr. Huppke,

Thank you for your recent submission BRAINCOM-2020-362
entitled "A novel remitting leukodystrophy associated with a variant in FBP2" to Brain Communications.

Your manuscript has been reviewed, and while the reviewers had largely positive comments, they have some constructive critiques that I think you could address in a minor revision of the manuscript. Therefore, I invite you to respond to the reviewer(s)' comments and revise your manuscript. The reviewer(s)' comments are included at the bottom of this message.

In addition to the reviewers' comments, please note our editorial comments added below to comply with our requirements for presentation of scientific data at Brain Communications.

Please format your revised manuscript according to our Instructions to Authors
https://academic.oup.com/braincomms/pages/General_Instructions – a summary of key points is attached to help with this.

To revise your manuscript, click the link below or log into https://mc.manuscriptcentral.com/braincom and enter your Author Centre, where you will find your manuscript title listed under "Manuscripts with Decisions." Under "Actions," click on "Create a Revision." Your manuscript number has been appended to denote a revision.

*** PLEASE NOTE: This is a two-step process. After clicking on the link, you will be directed to a webpage to confirm. ***

https://mc.manuscriptcentral.com/braincom?URL_MASK=b418ef91d8464e0bbc124359ee2f96ad

Please upload a revised manuscript with the changes you have made shown either with "track changes" or in coloured text.

When submitting your revised manuscript, you will be asked to respond to the reviewer(s) comments. Please be as specific as possible in your response to all of the comments.

The editorial team at Brain Communications also aims to increase transparency in scientific publishing and to demonstrate the value that peer review brings to the scientific process. Please therefore consider whether you are willing to have reviewer comments on your paper and your response published as supplementary data with the paper if it is accepted. This is entirely optional and will only be done if both the authors and reviewers agree. When submitting your revision you will have the opportunity to update the answer you entered previously.

IMPORTANT: Your original files are available to you when you upload your revised manuscript. Please delete any redundant files before completing the submission.

Because we are trying to facilitate timely publication of manuscripts submitted to Brain Communications, we would appreciate your revised manuscript as soon as possible. If it is not possible for you to resubmit within 3 months, the option to submit a revision will expire, and we would then consider your revised paper as a new submission. However, if you need a little more time, please get in touch and we can extend this deadline for you.

Once again, thank you for submitting your manuscript to Brain Communications and I look forward to receiving your revision.

Yours sincerely,
Dr. Jose Bras
Editor, Brain Communications

Reviewer(s)' Comments to Author:

Reviewer: 1

Comments to the Author
The work of Gizak et al. shows for the first time the association of mutations in FBP2 with the development of an Autosomal Dominant Leukodystrophy, an association concluded by familial

segregation with the disease, enzymatic function of the protein and analysis of patient fibroblasts. I believe the manuscript should be considered for publication with major revisions due to the lack of important quantifications in some results, nevertheless the amount of data provided is enough to conclude the association if quantification is provided. The fact that is a newly discovered association is also of major importance in the genetic diseases field.
Clinical description and genetic analysis are well characterized, and I would not recommend any changes, but experimental work should be improved mainly by providing quantifications to avoid any conclusion based on qualitative only analysis.
Major revisions:
Figure 5: I would suggest to please provide quantification of co-localization analysis when describing less or more co-localization in the results session. The total absence of co-localization of FBP2 with nuclei can be shown only qualitatively by co-staining with a nuclear marker. I suggest that co-localization with mitochondria should be properly evaluated. Mitochondrial polarization and ROS should also be quantified.
Figure 6: For the same reason, please provide co-localization analysis for Figure 6C and Figure 6E.

Minor comments: Please check word Co-localization or colocalization.

Reviewer: 2 (Franco Toni)

Comments to the Author
The authors describe a family with a dominant form of early-onset remitting white matter disorder affecting eight members in four generations. Detailed clinical history and data including longitudinal MRI are reported for three patients. In these patients, the disease manifested around one year of age with sudden global disability following mild febrile illness. Neurological symptoms improved with almost complete recovery within two years. Neuroimaging showed marked demyelination thta progressed for several months and was followed by remyelination. For other four patients, clinical history is not detailed and only MRI in adulthood is shown. Given the maternal transmission of the disease, mtDNA was investigated with no evidence of pathogenic sequence variants nor duplications/deletions. WES revealed the presence of a highly conserved missense variant p.Val115Met in the <i>FBP2</i> gene encoding muscle fructose 1,6-biphosphatase. The variant segregated with the disease being present in all affected members and absent in the only nonaffected member.
The authors performed several biochemical and cell biology experiments to support the pathogenic nature of the variant. In particular, they found that the variant 1) has a dominant negative effect on enzymatic activity and thermal stability of FBP2; 2) is associated with loss of FBP2 colocalization with mitochondria and nuclei, increased ROS production and abnormalities of the mitochondrial network.

Major comments:
1) the functional evidence for a pathogenic role of the <i>FBP2</i> variant is convincing. Biochemical and cell biology experiments are appropriately designed and performed
2) the study disclosed a prominent involvement of mitochondria as demonstrated by disturbances of mitochondrial network and ROS accumulation. Furthermore, plasma lactate was elevated in two patients and activities of respiratory chain complex II, III, and IV was reduced in muscle of one patient. Given the maternal transmission of the disease, the authors performed sequencing of the entire mtDNA and excluded the presence of mtDNA duplications and deletions. I believe that mtDNA studies should be completed by performing quantitative analysis in muscle in order to exclude mtDNA depletion.

Minor comment:
On p. 19, 1st par, lines 1-4: to avoid ambiguity, reference for GeneMatcher (Sobreira <i>et al.</i>, 2015) should not be placed at the end of the sentence but where "GeneMatcher database" is mentioned (line 1)

Editorial Office comments:

-Please supply a graphical abstract. A graphical abstract is a visual representation of the central finding or methodology of the paper. It will be displayed in the table of contents. Authors should strive to make the graphical abstract informative, interesting, visually appealing, and straightforward (no caption needed, no abbreviations except gene names). Please upload as a separate high resolution image file (tiff preferred). The technical requirements for the graphical abstracts are:
  --size of the submitted image: 1200 pixels square at 300dpi
  --font of the text: Arial 12-16 point (smaller fonts will not be legible online)

-Please add an Abbreviated Summary (up to 50 words) of your paper that captures the main purpose and conclusions of the work. The summary should state the main results written in the third person (‘[1st author surname] et al. report that …’) and conclusion. This summary will be used in the contents list online and should not contain any abbreviations other than those on the accepted abbreviations list

and accepted gene/protein names, making it accessible to the non-specialist. Please upload it as a Word document. Allowed abbreviations: AIDS; ANOVA; AMPA; ATP; A,T,C,G; CNS; CSF; CT; DNA; ECG; EEG; EMG; GABA; HIV; MRI; NMDA; PET; PCR; RNA.

-Please include a paragraph about data availability in the 'Materials and Methods' section. We strongly encourage data sharing either as supplementary information or uploads to repositories which you can link in the paper.

- To comply with our ethical standards, the appropriate checklist must be included (see Reporting Guideline section within 'Instructions to authors').

-Fig4b: please, replace green colour with magenta to make the figure color blind friendly and show single data points as n<10.

-Fig6: please, provide how many times the experiment was repeated and how many cells were considered/experiment.
