# Supplementary material for: A novel remitting leukodystrophy associated with a variant in FBP2
Source: Brain Commun. 2021 Mar 11;3(2):fcab036. doi: 10.1093/braincomms/fcab036 (PMC8097510; doi:10.1093/braincomms/fcab036)
Supplement: fcab036_Supplementary_Data [file fcab036_supplementary_data.zip › Review_files.zip!/Original Submission.pdf]

OXFORD UNIVERSITY PRESS | Brain Communications

A novel remitting leukodystrophy associated with a variant in FBP2

Journal:	*Brain Communications*
Manuscript ID	BRAINCOM-2020-362
Manuscript Type:	Original Article
Date Submitted by the Author:	20-Nov-2020

Complete List of Authors:	Gizak, Agnieszka; University of Wroclaw, Department of Molecular Physiology and Neurobiology<br>Diegmann, Susann; University Medical Center Göttingen, Pediatrics<br>Dreha-Kulaczewski, Steffi; University Medical Center Göttingen, Paediatrics and Paediatric Neurology<br>Wiśniewski, Janusz; University of Wroclaw, Department of Molecular Physiology and Neurobiology<br>Duda, Przemysław; University of Wroclaw, Department of Molecular Physiology and Neurobiology<br>Ohlenbusch, Andreas; University Medical Center Göttingen, Pediatric Neurology<br>Huppke, Brenda; University Medical Center Göttingen, Pediatric Neurology<br>Henneke, Marco; University Medical Center Göttingen, Pediatric Neurology<br>Höhne, Wolfgang; Charité – University Medicine Berlin, Institute of Biochemistry<br>Altmüller, Janine; University of Cologne, Cologne Center for Genomics (CCG)<br>Thiele, Holger; University of Cologne, Cologne Center for Genomics (CCG)<br>Nürnberg, Peter; University of Cologne, Cologne Center for Genomics (CCG)<br>Rakus, Darius; University of Wroclaw, Department of Molecular Physiology and Neurobiology<br>Gaertner, Jutta; University Medical Center Göttingen, Pediatric Neurology<br>Huppke, Peter; University Medical Center Göttingen, Pediatric Neurology

Keywords:	

SCHOLARONE™ Manuscripts

A novel remitting leukodystrophy associated with a variant in *FBP2*

Short title: A remitting leukodystrophy with a FBP2 variant

Agnieszka Gizak[1#], Susann Diegmann[2#], Steffi Dreha-Kulaczewski[2], Janusz Wiśniewski[1], Przemysław Duda[1], Andreas Ohlenbusch[2], Brenda Huppke[2], Marco Henneke[2], Wolfgang Höhne[3], Janine Altmüller[3], Holger Thiele[3], Peter Nürnberg[3], Dariusz Rakus[1]*, Jutta Gärtner[2]*, Peter Huppke[2]*

[#] contributed equally

* co-corresponding authors

Affiliations

[1] Department of Molecular Physiology and Neurobiology, University of Wrocław, 50-335 Wrocław, Poland.

[2] Department of Pediatrics and Pediatric Neurology, University Medical Center Göttingen, Georg August University Göttingen, Germany.

[3] Cologne Center for Genomics (CCG) and Center for Molecular Medicine Cologne (CMMC), University of Cologne, Faculty of Medicine and University Hospital Cologne, 50931 Cologne, Germany.

For Review Only

Corresponding authors:

Peter Huppke

Department of Pediatrics and Adolescent Medicine, Pediatric Neurology, University Medical Center Göttingen, Georg August University, Robert-Koch-Strasse 40, 37075 Göttingen, Germany. Fax: 0049551396252, Phone: 00495513913872

Jutta Gärtner

Department of Pediatrics and Adolescent Medicine, Pediatric Neurology, University Medical Center Göttingen, Georg August University, Robert-Koch-Strasse 40, 37075 Göttingen, Germany. 0049551396252, Phone: 0049551393085

Dariusz Rakus

Department of Molecular Physiology and Neurobiology, University of Wrocław, 50-335 Wrocław, Poland. Fax: 0048713222817; Phone: 0048713754053.

For Review Only

Abstract:

Leukodystrophies are genetic disorders of cerebral white matter that almost exclusively have a progressive disease course. We became aware of three members of a family with a disorder characterized by a sudden loss of all previously acquired abilities around one year of age followed by almost complete recovery within two years. Cerebral MRI and myelin sensitive imaging showed a pronounced demyelination that progressed for several months despite signs of clinical improvement and was followed by remyelination. Exome sequencing did not - identify any mutations in known leukodystrophy genes but revealed a heterozygous variant in the *FBP2* gene, c.343G>A, p.Val115Met, shared by the affected family members. Cerebral MRI of other family members demonstrated similar white matter abnormalities in all carriers of the variant in *FBP2*. The *FBP2* gene codes for muscle fructose 1,6-bisphosphatase (FBP2), an enzyme involved in gluconeogenesis that is highly expressed in brain tissue. Biochemical analysis showed that the variant has a dominant negative effect on enzymatic activity, substrate affinity, cooperativity and thermal stability. Moreover, it also affects the non-canonical functions of FBP2 involved in mitochondrial protection and regulation of several nuclear processes. In patients' fibroblasts, FBP2 shows no colocalization with mitochondria and nuclei leading to increased reactive oxygen species (ROS) production and a disturbed mitochondrial network. In conclusion, the results of this study indicate that the variant in *FBP2* disturbs cerebral energy metabolism and is associated with a novel remitting leukodystrophy.

Key words: Leukodystrophy, remitting, muscle fructose 1,6-bisphosphatase

For Review Only

Abbreviations:

ADC	apparent diffusion coefficient
ERAD	endoplasmic reticulum-associated protein degradation
ERLAD	ER-to-lysosomes-associated degradation
F-1,6-BP	fructose-1,6-bisphosphate
FBP1	fructose 1,6-bisphoshatase
FBP2	muscle fructose 1,6-bisphosphatase
HCF	heathy control fibroblasts

MRS	Magnetic Resonance Spectroscopy
MT	magnetization transfer
MTsat	MT saturation
ROS	reactive oxygen species
WM	white matter

Introduction

Leukodystrophies are a group of genetic disorders with primary involvement of the central nervous system white matter(van der Knaap and Bugiani, 2017). Most of these disorders have a progressive neurodegenerative course often leading to premature death. Four leukodystrophies with a remitting course have, however, recently been described: 1) Patients with variants in the *TMEM63A* gene, coding for a mechanosensitive ion channel, present with congenital nystagmus and motor delay in infancy associated with hypomyelination of the CNS (Yan *et al.*, 2019). During infancy the myelin deficit resolves, and the clinical course is favorable. 2) Variants in the *SLC13A3* gene, encoding the plasma membrane Na^+/dicarboxylate cotransporter 3, have been associated with a reversible leukoencephalopathy during febrile illness (Dewulf *et al.*, 2019). Clinical recovery was observed within days of the crisis and MRI changes normalized. 3) Recessive mutations in *GLIALCAM* cause a classical megalencephalic leukoencephalopathy with subcortical cysts. Autosomal dominant mutations in the same gene, however, have been associated with a remitting phenotype (Lopez-Hernandez *et al.*, 2011). 4) Finally, two families with mutations in *FDX2,* coding for a protein involved in mitochondrial [Fe-S] protein assembly, have recently been described in whom partial resolution of the leukoencephalopathy was reported but the clinical course remained progressive (Gurgel-Giannetti *et al.*, 2018).

In this article, we describe a family with a disorder that can also be classified as a remitting leukodystrophy. Affected family members presented with very pronounced white matter abnormalities that almost completely resolved over time, accompanied by remarkable clinical improvement. A missense variant of the *FBP2* gene coding for the muscle fructose 1,6-bisphosphatase (FBP2) segregates with the disorder. FBP is a regulatory enzyme of gluconeogenesis that catalyzes the hydrolysis of fructose-1,6-bisphosphate (F-1,6-BP) to fructose-6-phosphate (F-6-P) and inorganic phosphate. In contrast to the liver isozyme, fructose

For Review Only

1,6-bisphoshatase (FBP1), which regulates glucose production in the liver, kidney and jejunum, FBP2 expression is not restricted to gluconeogenic tissues but is ubiquitous (Al-Robaiy and Eschrich, 1999). Crystallographic studies have demonstrated that FBP2 is a homotetrameric protein which may exist in two quaternary conformations: an active cross-like structure R-state, and an inactive T-state (Barciszewski *et al.*, 2016). Allosteric inhibitors such as AMP and NAD^+ stabilize the tetrameric conformation of FBP2 and induce a shift towards the T-state (Barciszewski *et al.*, 2016). The capability of FBP2 to adopt various quaternary conformations determines its potential to interact with different cellular binding partners and assume different cellular roles depending on the metabolic and physiological conditions.

During the last decade it has been shown that FBP2 has also acquired additional functions as a moonlighting protein. The non-canonical functions include FBP2 association with mitochondrial proteins in GSK3β- in a calcium-dependent manner, thereby making the protein instrumental in the protection of organelles against stress stimuli (Gizak *et al.*, 2012). Furthermore, FBP2 is known to localize in nuclei where it is involved in the regulation of cell mortality and survival. Biochemical and clinical evidence suggests that the variant of FBP2 detected in the family described in this article acts in a dominant negative fashion, affecting both enzymatic activity and the non-canonical functions of the enzyme.

For Review Only

Methods

DNA samples were obtained following informed consent according to the Declaration of Helsinki and approval by the ethic commission from the University Medical Center Göttingen, Göttingen (approval number 2516). Fibroblasts from affected individuals used in this study were obtained from skin biopsies previously sampled for routine diagnostic testing.

Proton Magnetic Resonance Spectroscopy

Our multimodal MR-protocol included single-voxel proton Magnetic Resonance Spectroscopy (MRS). Proton MR spectra (64 accumulations) were acquired with use of a STEAM localization sequence with repetition time /echo time/mixing time = 6000/20/10 ms as described (Frahm *et al.*, 1989). Studies older than 15 years were conducted at 2 T (Magnetom Vision; Siemens Medical Solutions, Erlangen, Germany) all others at 3 T (Magnetom Trio, TIM TRIO and Prisma Fit; Siemens Healthcare). The 4.85 ml (2 T) and 4.1 ml (3 T) volumes-of-interest

were placed within the parieto-occipital white matter (left or right) covering structural lesions. Absolute concentrations of lactate were determined by LCModel (Provencher, 1993). Normal ranges were taken from Pouwels et al. and our own data base (Pouwels *et al.*, 1999).

Magnetization Transfer-Imaging

Whole brain magnetization transfer (MT)-imaging, as another part of our myelin specific MR-imaging protocol, was performed at 3 T (Magnetom TIM TRIO and Prisma Fit) using a 3D FLASH sequence with 1.25 mm isotropic resolution and 240 mm field-of-view. MT contrast was imposed upon a proton density -weighted reference (TR/TE/α = 25/4.9 ms/5°, using partial acquisition techniques) by applying a 12.8 ms Gaussian MT-pulse of 540° nominal flip angle 2.2 kHz off resonance prior to excitation. By means of a second T1-weighted reference (TR/α=11 ms/15°, 1.5 minutes), maps of the percentage MT saturation (MTsat) were calculated, a semi-quantitative MT-parameter as described in Helms et al (Helms *et al.*, 2008b). Data processing was scripted using the routines of the FSL 4.1 software library of the Center for Functional Magnetic Resonance Imaging of the Brain (FMRIB, Oxford, UK, www.fmrib.ox.ac.uk/fsl) (Helms *et al.*, 2008a, 2010). The blue-gray-red-yellow color scale of the MTsat maps covered a range from -0.1 pu (light blue; lower limit of the CSF mode) to 1.2 pu (gray, gray matter) to 2.5 pu (yellow; fully myelinated white matter)(Dreha-Kulaczewski *et al.*, 2012).

Sequencing of mitochondrial DNA

Genomic DNA was extracted from peripheral blood from patient III.5 using standard techniques. Two overlapping mtDNA fragments of 11 and 9 kb encompassing the entire mitochondrial genome were generated with the Expand Long Template PCR System (Roche, Mannheim, Germany) according to the recommendations of the manufacturer. Sanger sequencing was performed using the BigDyeTM Terminator v3.1 Cycle Sequencing chemistry on an Applied Biosystems 3500 Genetic Analyzer platform (Applied Biosystems, Darmstadt, Germany). Analyzed sequences were compared with reference NCBI data, accession number NC_012920.1. Primer sequences as well as PCR and sequencing conditions are available on request.

For Review Only

Whole Exome Sequencing

Whole exome sequencing was performed on DNA from EDTA blood of six affected family members (II.2, II.4, III.2, III.4, III.5, IV.1, Figure 1). Each Sequencing barcoded library was prepared using the Agilent SureSelect V6 enrichment Kit. Purified and quantified library pool was subsequently sequenced on an Illumina HighSeq 4000 sequencing instrument (Illumina, San Diego, CA, USA) using a paired end 2x 75bp to 2x 101bp protocol and an allocation of 6 exomes per lane. Data processing, analysis and filtering were performed using the ‚Varbank 2' GUI and pipeline version 3.4/3.5 (CCG, University of Cologne, Germany). Reads were mapped to the human genome reference build GRCh38 using the BWA-MEM alignment algorithm. GATK HaplotypeCaller, Samtools mpileup and Platypus variation calls were filtered for high-quality (QD>5; ARF>0.25; MQ>50; FS<40; MQRankSum>-5; ReadPosRankSum>-5; passed VQSR filter) rare (MAF ≤ 0.005 based on the maximum observed population allele frequency in gnomAD v2) variants, predicted to modify a protein sequence or to impair splicing, implicated by reduced maximum entropy scores (MaxEntScan)(Li *et al.*, 2009; McKenna *et al.*, 2010; Rimmer *et al.*, 2014).

For detection of the common genetic cause of all affected family members the dataset was filtered for rare common genetic variants. False positive and irrelevant variants were further reduced by taking advantage of our InHouseDB. The pathogenicity of all received variants was evaluated using several *in silico* analysis tools by the dbNSFP/dbscSNV version 3.4 database.

Validation of variant of interest in *FBP2* as well as co-segregation analysis within the family was performed using Sanger sequencing.

For Review Only

FBP2 expression, mutagenesis and fluorescent labeling

Human FBP2 mutant V115M was created using the procedure described earlier. The following primer pairs were used: FW: AAG AGG GGG AAA TAC ATG GTC TGC TTT GAC C; RV: GGT CAA AGC AGA CCA TGT ATT TCC CCC TCT T. Sequences were confirmed by DNA sequencing using T7 promoter and T7 terminator primers.

WT-FBP2 and V115M-FBP2 were isolated according to Wisniewski et al[9]. Both FBP2 variants were labeled using FITC as described previously (Wisniewski *et al.*, 2017).

Enzyme activity measurements

FBP2 activity was measured at pH 7.5 and 37°C using a glucose 6-phosphate isomerase—glucose 6-phosphate dehydrogenase coupled assay and the reduction of $NADP^+$ to NADPH

was monitored spectrophotometrically at 340 nm (Zarzycki *et al.*, 2011). Parameters were estimated in MATLAB's curve fitting tool, using nonlinear least squares regression with trust-region algorithm and bisquare robust fitting.

FBP2 inhibition by AMP was measured in triplicate for each concentration of the ligand.

Protein melting assay

Protein precipitation was assessed by measurement of light scattering at 600 nm wavelength using an Agilent 8453 UV–Vis spectrophotometer with 10 mm light path length. The measurements were conducted at temperatures ranging from 35°C to 80°C in 2°C steps. Samples were equilibrated for 2 min before each measurement. To avoid sedimentation, the samples were stirred at 200 rev min^{-1}. Buffer and protein concentration were the same as in the enzymatic activity measurements. 300 μM AMP was added for T-state measurements. To determine the melting temperatures, derivative curves of absorbance versus temperature were calculated. The maxima of the derivative curves correspond to the melting temperatures.

Cell culturing

Fibroblasts were cultured as we described before (Duda *et al.*, 2020).

Fluorescent In Situ Hybridization (FISH)

FISH was performed as described in (Mamczur *et al.*, 2015). The following oligonucleotides complementary to human mRNA sequences were used: WT-FBP2 (5′-(Cyanine3) CCT CAG GGA ATT TCT TTT TCT GCA CAT ATC CAG TGG TGG C-3′), V115M-FBP2 (5′-(Cyanine5) CCA GTG GGT CAA AGC ACA TCA CGT ATT TCC CCC GCT TCT C-3′). All the oligonucleotides were synthesized by Merck KGaA. In controls, the oligonucleotide probes were omitted.

Immunocytochemistry

Cells growing on coverslips were fixed in 4% paraformaldehyde, permeabilized with 0.1% Triton X-100 in PBS and incubated with 3% BSA in PBS to reduce nonspecific binding of antibodies. Then they were incubated with the respective primary antibody: mouse monoclonal to PSMC6 (1:500, Abcam, Cambridge, UK, ab22639), mouse monoclonal to LAMP-1 (1:500, Santa Cruz Biotechnology, Dallas, TX, USA, sc-20011), rabbit polyclonal to FBP (1:500, purified and characterized as described in (Gizak and Dzugaj, 2003)).

For Review Only

The primary antibodies were then detected using fluorophore-labeled secondary antibodies: goat polyclonal to mouse IgG (AlexaFluor488) (1:1000; Abcam, Cambridge, UK, ab150113), goat polyclonal to mouse IgG (AlexaFluor633) (1:1000; ThermoFisher Scientific, Waltham, MA, USA, a21050), goat polyclonal to rabbit IgG (AlexaFluor488) (1:1000; ThermoFisher Scientific, Waltham, MA, USA, a11034), goat polyclonal to rabbit IgG (AlexaFluor633) (1:1000; ThermoFisher Scientific, Waltham, MA, USA, a21070). To visualize mitochondria, the cells were counterstained with MitoTracker Deep Red (ThermoFisher). In the controls, the primary antibodies were omitted.

Images were acquired using FV-1000 confocal microscope (Olympus) with ×60 (oil, Plan SApo, NA = 1.35) objective using the Sequential scan option. The quantification of fluorescence signal was performed using the ImageJ software (Schneider *et al.*, 2012). The cells were marked and the mean fluorescence within the marked areas was measured. The measurements were taken from at least 50 cells from randomly selected areas. Results are expressed as a mean and standard deviation. If not stated otherwise, data were checked for normality using the Shapiro–Wilk test, for equality of variations using the F-test, and for the evaluation of statistical significance the two-tailed Student's T-test was used. A probability of $p < 0.05$ was considered to represent a significant difference. All the experiments were done at least in triplicate.

For Review Only

Measurements of cellular ROS production and mitochondrial polarization

Cellular ROS production was measured using dihydrofluorescein diacetate (H2DCF-AC) as described in (Gizak *et al.*, 2020). Briefly, live cells were loaded with 5 µM H2DCF-AC (20 min, 37°C), rinsed with Hank's Balanced Salt Solution, and mounted on slides. The fluorescence of the dye was excited at 488 nm for 500 ms. Mitochondrial ROS production was determined by MitoTracker Red CM-H2XRos (ThermoFisher) that fluoresces only upon oxidation.

Loss of the mitochondrial membrane potential was detected using JC-1 fluorescent dye (Mitochondrial Permeability Transition Detection Kit, AbD Serotec) according to the manufacturer's instruction. Polarized mitochondria accumulate more of the dye and are red. In cells with depolarized mitochondria, most of the dye is dispersed in cytoplasm and has green fluorescence. The ratio of red to green fluorescence reflects the degree of the mitochondrial membrane polarization. The JC-1 dye was excited at 488 nm for 500 ms and the emission was observed using a long pass filter, which allowed for simultaneous observation of green (monomers) and red (aggregates) fluorescence.

Protein delivery to fibroblasts and assessment of ERAD activity

Healthy fibroblasts were grown on coverslips. FITC-labeled FBP2 forms were delivered into the cells using CHARIOT Protein Delivery Reagent (Active Motif) as we described before (Wisniewski *et al.*, 2017). Then the cells were counterstained with MitoTracker Deep Red FM or with ER-Tracker Blue White (ThermoFisher) and examined under confocal microscope; in the images, they are shown in red. To test the endoplasmic reticulum-associated degradation of the V115M-FBP2 mutant, healthy fibroblasts were pre-incubated for 1 h with 8 μM Eeyarestatin, the inhibitor of ERAD (endoplasmic reticulum-associated protein degradation), and transfected with the mutant form of FBP2 in the presence of the inhibitor.

Co-immunoprecipitation

In the co-immunoprecipitation experiment, the cell lysates obtained from cell cultures were used. Briefly, the lysates (100 μg protein) were mixed and incubated overnight at 4°C with 50 μg/mL of antibodies against ubiquitin (Lys48-specific, Merck KGaA, Darmstadt, Germany, 05-1307). The complexes were precipitated using 200 μL/mL of the Protein G Agarose (Merck). The precipitates were centrifuged at 4000× g for 2 min and washed with PBS. In control reactions, the precipitating antibodies were omitted. The precipitates were then resuspended in the Laemmli's buffer, resolved by SDS–PAGE, and Western Blot analyses were performed with the use of primary antibodies detecting FBP (isolated and purified as described in (Gizak and Dzugaj, 2003)).

For Review Only

Results

Case reports:

The four-generation pedigree is shown in Fig. 1. Case reports and description of the MRI findings of the family members who did not present with a crisis at age one year are included in figure legends 1 and 2 respectively.

Case reports of the three patients with a remitting leukoencephalopathy in infancy

Patient III.2, female, was born at term after an uneventful pregnancy. Psychomotor development was normal until age 13 months when, during a bronchitis episode with fever, she developed a paresis of the left leg followed within days by the loss of almost all abilities. At presentation to hospital she was irritable, socially withdrawn, unable to sit, crawl or stand and

displayed only minimal motor activity. Significant feeding difficulties made tube feeding necessary for 4 weeks. On examination muscle tone in the legs was increased, while otherwise decreased. First cerebral MRI was performed at age 14 months and showed a leukodystrophy affecting most of the supratentorial white matter with sparing of the periventricular and infratentorial white matter (Fig. 2A). Over the next 11 months she made a slow but continuous recovery. At age 16 months she was able to fixate and hold objects again but was still unable to sit unsupported. By age 24 months no remaining abnormalities were found on neurological examination. Consistent with the clinical findings, a drastic improvement in white matter abnormalities was seen on cerebral MRI performed at age 23 months. The patient is now 25 years old and works as a kindergarten teacher. Her only residual deficit is a very mild gait abnormality. Only residual gliotic lesions affecting the corpus callosum and periventricular white matter were seen on cerebral MRI at age 20 years (Fig. 2C).

Patient III.5, female, was born after an uneventful pregnancy and developed normally until age 10 months when all abilities were rapidly lost during a febrile illness. Like patient III.2, she was very irritable, showed no social contact and refused to eat necessitating tube feeding for four months. Muscle tone in the legs was increased, while otherwise decreased. Cerebral MRI at age 11 months also showed a leukodystrophy with pronounced signal changes affecting the deep cerebral white matter and to a lesser extent the nucleus dentatus (Fig. 2D). Over the following 20 months a slow but continuous improvement in her condition was observed. At age 17 months she was able to sit unsupported again and speak single words, at 21 months she could walk short distances and speak 2-word sentences and at 30 months her only reported deficit was an inability to run fast. In contrast to the positive clinical development, MRI imaging initially showed a further progression of white matter abnormalities until age 21 months (Fig. 2E) before gradually almost completely resolving. Between age 8 and 10 years four focal seizures were reported. Now at age 24 years she works as a social worker, has a slight gait abnormality and avoids walking long distances. Only residual white matter changes remain on cerebral MRI (Fig. 2G).

Patient IV.1, female, daughter of patient III.5, was born after an uneventful pregnancy at term. After an initial normal period of development, her ability to sit, crawl and walk with support was lost within two days coinciding with a viral infection at age 12 months. She was very irritable, lying with extended, crossed legs. As in the two previous cases, the acute episode was followed by a gradual recovery of lost abilities. At 18 months she was able to sit unsupported again, crawl slowly and stand with support. When last seen at age 21 months she was walking short distances unsupported but speaking only a few single words. Cerebral MRIs obtained over

For Review Only

a follow-up period of 16 months displayed a pattern of white matter abnormalities that resemble those seen in patients III.2 and III.5 (Fig. 2H-K). T2 hyperintensities were most pronounced in the centrum semiovale with a central strand of more normal appearing white matter. Interestingly, the apparent diffusion coefficient (ADC) of the initial MRI study performed shortly after onset of symptoms revealed widespread distinctly restricted diffusion in the T2 lesional areas (Fig. 2L). In follow up studies the ADC signal then reversed to bright intensities indicating enhanced diffusion (Fig. 2M-O).

None of the patients displayed any dysmorphic features and somatic growth including head circumference was normal. In all three patients an extensive diagnostic work-up was performed. Analysis of plasma lactate in the acute stage was normal in patient III.2 but elevated in patient III.5 (3.8 mmol/l) and patient IV.1 (3.5 mmol/l) (reference 0.5-2.2 mmol/l). Normal values were seen for lactate in CSF (III.2, III.5, IV.1), CSF Protein (III.2, III.5, IV.1), copper, ceruloplasmin (III.2, III.5), carbohydrate-deficient transferrin (III.5, IV.1), carnitine and acylcarnitine (III.5, IV.1), very long chain fatty acids (III.2, III.5, IV.1), enzyme activities of arylsulfatase A, beta-galactosidase, hexosaminidase A, hexosaminidase B, galactosylceramidase (III.2, III.5, IV.1), amino acids in CSF and plasma (III.5, IV.1) and organic acids in urine (III.5).

Biopsy of skin and muscle from patient III.5 was performed. Activities of pyruvate dehydrogenase complex and respiratory chain enzymes in fibroblasts showed moderately reduced activities of complex III and complex IV in fibroblasts and reduced activities of complex II, III and IV in muscle. Muscle histology showed no abnormalities, in particular no ragged red fibers.

Nerve conduction velocity (III.2, III.5, IV.1) as well as acoustic evoked potentials (III.2, III.5, IV.1) were repeatedly normal. Visual evoked potentials were normal in III.2 and abnormal in III.5 and IV.1 in the acute phase.

Proton MR-spectroscopy and Myelin sensitive imaging indicate mitochondrial failure and demyelination during the acute phase and subsequent recovery

Localized MRS performed in Patients III.2, III.5 and IV.1 shortly after onset of symptoms uniformly revealed lactate elevation in white matter lesions in all three patients. In patient III.5 and her daughter, patient IV.1 follow up MRS were obtained over a period of 14 and 1.3 years

For Review Only

respectively. Results demonstrated a gradual normalization coinciding with the clinical improvement (Fig. 3A).

MTsat maps obtained in IV.1 depicted initial myelin deficit most pronounced in the lesions (blue color-coded white matter (WM) areas, Fig. 3B-D). In comparison to the maps of a younger healthy control (age 1.5 years) in Fig. 3 E the myelin loss appeared more widespread than depicted by conventional T2 weighted imaging (Fig. 2H-K). Subsequent studies six months and 1.3 years later indicated progressive demyelination (Fig 3C) followed by remyelination (appearance of gray-red-orange-yellow color-coded WM, Fig 3D)

Exome sequencing detects a variant in FBP2 with unknown pathogenicity

Because segregation within the family was compatible with maternal inheritance, the complete mitochondrial genome from patient III.5 was sequenced. No variants with known or suspected pathogenicity were detected. Furthermore, no deletions or duplications of mitochondrial DNA were seen.

Exome sequencing was initially performed in DNA samples from family members, III.2, III.5 and IV.1 and later extended to II.2, II.4, III.4 (Supp. Tab. 1). No mutations in known leukodystrophy genes were detected but the analysis revealed heterozygous missense variants common in all patients in *CCT6A* (c.446T>C, p.Ile149Thr) and *FBP2* (c.343G>A, p.Val115Met) (Supp. Tab. 2). The variant in *CCT6A* is a frequent multiallelic site in gnomAD and is predicted to have a benign impact (Supp. Tab. 2). Therefore, it was not considered pathogenic. The variant p.Val115Met in *FBP2* is not described in our in-house population database and only once in a European male in gnomAD (accessed in May 2020) (Allele frequency: 0.000003979). The *FBP2* variant p.Val115Met is predicted to be possibly damaging by 10 out of 13 *in silico* analysis tools (Supp. Tab. 3). Furthermore, the position Val115 is highly conserved both in vertebrate FBP2 and FBP1, and also in FBPs of insects and plants, as can be derived from a multiple sequence alignment and is highly conserved in terrestrial vertebrates (Supp. Fig. 2A). Val115 is situated in a hydrophobic pocket formed by the residues Val94, Tyr140, Ala153. Active site and metal binding sites are not directly involved but an exchange of the small valine side chain by the much larger methionine may disturb the structure locally (Suppl. Fig. 2B). Sanger sequencing was performed in all family members who agreed to the analysis (Suppl. Fig. 1A). The *FBP2* variant was confirmed in the family members who had undergone exome sequencing and was also confirmed in patient IV.2 who has subsequently

For Review Only

been shown to have leukoencephalopathy, developmental delay and behavioral problems. It was also detected in patient III.3 who has a history of psychiatric problem but declined MR imaging (Suppl. Fig. 2). The *FBP2* variant was not detected in the only healthy offspring, III.1. As the genetic results suggest a causative role of the *FBP2* c.343G>A, p.Val115Met variant in the disorder seen in the family we conducted biochemical analyses to support its pathogenicity.

The V115M-FBP2 variant affects activity, substrate affinity and thermal stability of FBP2

The residue 115 is not directly involved in the mechanism of catalysis and allosteric inhibition of FBP2. However, the analysis of kinetic properties of the recombinant protein demonstrated that V115M-FBP2 was about two-fold less active than WT-FBP2 (Fig. 4A), and its affinity to the substrate, F-1,6-BP, was over 1.5 times lower. V115M-FBP2 was also significantly less sensitive to the action of the allosteric inhibitor AMP and the cooperativity of the inhibition was lost. Since the cooperativity of the allosteric inhibition of FBP2 is linked to the mechanism of R-to-T transition and tetramerization, the V115M mutation most likely disrupts the quaternary structure of the protein. Comparison of melting points of V115M-FBP2 and WT-FBP2 using the thermal denaturation method showed that, as in the case of WT-FBP2, the presence of AMP during incubation improved the thermal stability of V115M-FBP2 (Fig. 4B). However, both the AMP-saturated and the AMP-free V115M-FBP2 was significantly less stable than WT-FBP2. To verify these differences, we monitored activities of both enzyme variants during incubation at 37°C. Their activities decreased about two-fold after the first 20-25 hours of incubation, however, during the subsequent 25 hours, activity of WT-FBP2 remained unchanged while activity of V115M-FBP2 was constantly decreasing (Fig. 4C).

For Review Only

The V115M-FBP2 variant alters subcellular localization of FBP2

Next, we analyzed the subcellular localization of FBP2 in healthy fibroblasts, and fibroblasts derived from patient III.5. As expected, in heathy control fibroblasts (HCF), FBP2 was localized in cytoplasm where it was relatively homogeneously dispersed in the whole cell. A slightly fibrillar localization of the enzyme might be attributed to co-localization of a fraction of the protein with mitochondria (Gizak *et al.*, 2012). The enzyme was also present in nuclei (Fig. 5A). In contrast, in fibroblasts derived from patient III.5, FBP2 showed granular

localization. It was present only within the cells' bodies, not in protrusions, and did not localize in cells' nuclei.

The V115M-FBP2 variant does not colocalize with mitochondria, correlating with disturbance of their network and increase in ROS production

The localization of FBP2 in cytoplasm seen in fibroblasts from patient III.5, suggested mitochondrial accumulation of the enzyme, but immunostaining revealed that in fact, it did not co-localize with mitochondria, even if the cells were treated with GSK3β inhibitor (Fig. 5B) or elevated Ca^{2+} concentrations (data not shown) which have been shown to stimulate accumulation of FBP2 on mitochondria (Gizak *et al.*, 2012). Both factors increased colocalization of the FBP2-related fluorescence with mitochondria in HCF. To conclusively demonstrate that the lack of FBP2 colocalization with mitochondria is a result of inability of V115M-FBP2 to interact with the organelles we transfected HCF with FITC-labeled V115M-FBP2 and found no accumulation of the FITC-related fluorescence on mitochondria (Fig. 5C).

Next, we studied the mitochondrial network in fibroblasts from patient III.5. We found that the network was less developed and mitochondrial membranes were less polarized compared to HCF (Fig. 5D). Moreover, the disturbances in the mitochondrial network were accompanied by an increased production of ROS, both in cytoplasm and in mitochondria.

The V115M-FBP2 variant is degraded by ERAD and ER-to-lysosomes-associated degradation (ERLAD) pathways

Surprisingly, the total fluorescent signal associated with polyclonal antibodies against FBP2 was much stronger in fibroblasts from patient III.5 than in HCF (Fig. 6A). This raised the question, if the elevation of FBP2-related signal was a result of increased expression of V115M-FBP2 and/or WT-FBP2. Since antibodies against FBP2 do not discriminate between these two variants we used fluorescently labeled oligonucleotides against both forms of FBP2 to quantify their expression. As expected, expression of V115M-FBP was only seen in fibroblasts from patient III.5, but not in HCF, demonstrating specificity of the oligonucleotides (Fig. 6B). Moreover, in fibroblasts from patient III.5, the expression of WT-FBP2 was increased. This prompted us to analyze the colocalization of WT-FBP2 and V115M, with various systems of protein turnover in a cell. We found that transfection of wild type fibroblasts with FITC-labeled

For Review Only

V115M-FBP2 and WT-FBP2 led to colocalization of the mutant protein, but not the WT-FBP2, with the endoplasmic reticulum (ER) (Fig. 6C) ER is a part of the cellular machinery of misfolded protein degradation. Endoplasmic-reticulum-associated protein degradation (ERAD) is a process in which proteins are dislocated from ER to the cytosol for ubiquitylation and proteasomal degradation. Treatment of fibroblasts with the ERAD inhibitor Eeyrestatin decreased the level of FITC-labeled V115M-FBP2 colocalization with ER (Fig. 6C). In line with this, we observed a higher level of FBP2 (the sum WT-FBP2 and V115M-FBP2) polyubiquitylation in fibroblasts from patient III.5 compared to HCF (Fig. 6D). We also found that most of the V115M-FBP2-fluorescence colocalized with PSMC6 protein which is a component of the 26S proteasome, although the total level of PSMC6 in these cells was not elevated (Fig. 6E). It is known that some defective proteins do not engage ERAD machinery because their aggregates are too large and/or cannot be relocated across the ER membrane (ERAD-resistant misfolded proteins)(Houck *et al.*, 2014). The low thermal stability of the V115M-FBP2 and the presence of the enzyme in granule-like structures, suggested that the protein may be only partially eliminated by ERAD. Instead, it may be predominantly cleared by the alternative, ERAD-independent process of misfolded proteins degradation referred to as ELRAD (ER-to-lysosome-associated degradation), involving ER-phagy, microautophagy or vesicular transport (Fregno *et al.*, 2018). One of the indicators of the ELRAD process intensity is the abundance of lysosome-associated membrane glycoprotein 1 (LAMP-1) which represents the major constituent of lysosomal membrane proteins in a cell (Fregno *et al.*, 2018). Results of our study showed that the expression of LAMP-1 protein was over 2.5-times higher in patient III.5 fibroblasts than in HCF (Fig. 6F). This points to ELRAD as the main system involved in the clearance of defective variant of FBP2.

For Review Only

Discussion

In this article, we describe a family with a novel white matter disease. The three index patients experienced a severe crisis during a febrile illness at age 10-13 months resulting in a loss of all learned abilities. The acute episode was followed by a gradual recovery phase and achievement of normal developmental status within 1-2 years. Little is known about the early childhood of patients II.1 and II.2, as both grew up in an orphanage because their mother was unable to care for them due to a psychiatric disorder. However, of note is that they both display a similar residual MRI pattern to their daughters. A further family member, patient III.4, also has documented reversible white matter changes on repeated MRI imaging. Remarkably, none of

the index patients has experienced a second attack indicating that there might be a phase of vulnerability in brain development around the end of the first year of life.

Cerebral MRI at the time of the crisis revealed dramatic leukodystrophy of the periventricular white matter and the corpus callosum, more severe than in any of the known remitting leukodystrophies. White matter changes in the T2 weighted images were seen to increase for several months despite clinical improvement. In patient IV.1 DWI, myelin imaging and MRS were performed. DWI in the initial acute phase showed restricted diffusion in the affected white matter indicating cytotoxic edema consistent with a metabolic crisis. This finding was supported by MRS evidence of increased lactate in the white matter. During the following months diffusion was temporarily increased compatible with a reduction of myelin and subsequent remyelination. Consistently, myelin imaging initially demonstrated a massive decrease followed by a gradual increase and almost normalization of white matter myelin. The imaging data together with the clinical course are best compatible with an event associated with massive demyelination followed by remyelination over a period of 1-2 years. The near complete recovery makes preferential neuronal damage unlikely. We therefore hypothesize that the primary affected cells in this disorder are the oligodendrocytes.

Following exclusion of mutations in the mitochondrial DNA and in known leukodystrophy genes, exome sequencing resulted in the detection of only one variant that segregated with the leukodystrophy in the family and not frequently reported in current databases; *FBP2* c.343G>A, p.Val115Met. The affected amino acid Val115 is highly conserved in terrestrial vertebrates and is predicted to be damaging. In all available family members, the variant segregated with leukoencephalopathy on Sanger sequencing of the *FBP2* gene. Subject III.1 is the only offspring who carries the WT allele. She has no known neurological disorder and normal cranial MR imaging (data not shown).

So far, no human disorders have been linked to variants in the *FBP2* gene. Mutations in *FBP1*, coding for FBP1, a key enzyme of gluconeogenesis that is expressed in liver and kidney, cause fructose-1,6-bisphosphate deficiency, a disorder characterized by recurrent severe metabolic crises after fructose ingestion or during febrile illness. To assess the pathogenicity of the V115M-FBP2 variant, we investigated enzymatic function, in particular the kinetic properties and stability of the variant. We found that V115M-FBP2 has reduced activity, thermal stability and substrate affinity. Moreover, the enzyme was less sensitive to the action of allosteric inhibitor AMP and the cooperativity of the inhibition was lost. Under physiological conditions FBP2 forms dimers and tetramers, thus we speculate that WT-FBP2 and V115M-FBP2 co-

For Review Only

expressed in a cell form heteromers with reduced enzyme activity and stability explaining a dominant negative effect of the variant. The role of gluconeogenesis in the brain, where FBP2 but not FBP1 is expressed, is not fully understood but there is evidence that it is an important part of the local energy metabolism under physiological and pathological conditions (Yip *et al.*, 2016). In fructose-1,6-bisphosphate deficiency of the liver enzyme FBP1, acute attacks with hypoglycemia and lactic acidosis are often triggered by febrile infections. In the 3 index patients described here, the severe crisis also followed a febrile infection and lactate was elevated in the white matter during the acute phase on MRS. These findings indicate that defective gluconeogenesis might contribute to the pathophysiology. As synthesis of myelin is a very energy consuming process the oligodendrocytes might be particularly vulnerable to such a defect during the phase of intense myelination at the at the end of the first year of life (Harris and Attwell, 2012).

On fibroblast analysis, WT-FBP2 was increased in the fibroblasts of patient III.5. Presumably, this was due to reduced FBP2 function in the cell, and subsequent upregulation of FBP2 expression. We were also able to demonstrate preferential clearing of the defective protein by ERLAD leading to upregulation of LAMP-1 expression. These findings further support pathogenicity of the variant.

As recently reviewed by Huangyang et al., insights from cancer research have shown that many metabolic enzymes have activities outside of their established metabolic roles, including the regulation of gene expression, DNA damage repair, cell cycle progression and apoptosis (Huangyang and Simon, 2018). The authors speculate that modulating gene expression by metabolic enzymes facilitates adaptation of the cell to rapidly changing environments. A recently described example of a genetic defect that only affects the non-canonical functions of a metabolic enzyme involves the HK1 gene. Autosomal recessive mutations in the HK1 gene, coding for hexokinase 1, lead to a loss of enzymatic function and cause non-spherocytic hemolytic anemia. However, heterozygous de novo missense mutations in the same gene detected recently in six patients with developmental delay, intellectual disability, structural brain abnormality, and visual impairment, were associated with normal hexokinase avtivity (Okur *et al.*, 2019).

FBP2 reportedly also has several other functions in addition to enzymatic activity. Firstly, an involvement in mitochondrial protection has been shown. Upon activation of glycolysis by insulin, FBP2 colocalizes with mitochondria resulting in a reduced rate of calcium-induced mitochondrial swelling and protection of ATP synthesis (Gizak *et al.*, 2012). We analyzed the

For Review Only

effect of the V115M-FBP2 variant on mitochondria in fibroblasts of patient III.5 and found a pronounced disturbance of the mitochondrial network. In fact, the V115M-FBP2 variant did not colocalize with mitochondria, and ROS production in the organelles and whole cell was increased. Also supportive of mitochondrial dysfunction was evidence of elevated lactate levels in the white matter during the acute attack and subsequent normalization over the following months coinciding with clinical improvement. Interestingly, the MRI pattern seen in our patients matches that seen in Kearns-Sayre or Leigh syndrome, both classical mitochondrial disorders (van der Knaap, 2005). However, such patients have a progressive disease course with grey matter pathology especially of the basal ganglia which was not seen in the patients described here. In conclusion, while we collectively have evidence suggesting the pathophysiology of the disorder described in this article involves mitochondrial dysfunction, it does not, however, resemble a classical mitochondrial disorder.
A further function attributed to FBP2 is regulation of nuclear processes. Like many glucose metabolism enzymes, FBP2 has a classical Nuclear Localization Sequence (Gizak *et al.*, 2009). FBP2 interacts with histone and non-histone nuclear proteins and has been found to restrain mitochondrial biogenesis and respiration by colocalization with the c-Myc transcriptions factor (Mamczur *et al.*, 2012; Huangyang *et al.*, 2020). Furthermore, in cardiomyocytes and cancer cells, FBP2 has a recognized role in the regulation of the cell cycle (Gizak *et al.*, 2006). Immunostaining patient III.5 fibroblasts we were not able to detect FBP2 in nuclei. This suggests a dominant negative effect of V115M-FBP2 on nuclear localization, presumably due to heteromerization of WT and mutant proteins, and indicates that the nuclear functions of FBP2 are diminished in *FBP2* related-leukodystrophy.

We conducted a search using the GeneMatcher database for further families with a similar phenotype and variants in the *FBP2* gene and inquired with other international specialists in leukodystrophies if they had seen similar cases but were so far unsuccessful (Sobreira *et al.*, 2015). It is possible that only variants that affect both the enzymatic function and the non-canonical functions of FBP2 will be associated with the clinical phenotype seen in the family described here. Furthermore, FBP2-related leukodystrophy is, no doubt, an extremely rare disorder and misinterpretation as an autoinflammatory disorder such as acute disseminated encephalomyelitis, the initial diagnosis in patients III.2 and III.5, may hamper recognition of further cases.

In conclusion, we report on a novel disorder that we termed *FBP2*-related leukodystrophy. The phenotype is characterized by a metabolic crisis at the end of the first year of life leading to widespread demyelination and loss of abilities. The patients recover over several months

For Review Only

regaining normal development accompanied by remyelination. The variant in *FBP2* that was found to segregate in the family with the disorder, damages not only the enzymatic activity but also the non-canonical functions of FBP2 involved in mitochondrial protection and nuclear localization. FBP2-related leukodystrophy is a novel member of the group of remitting leukodystrophies.

Funding: This work was supported by the German Research Foundation (DFG, Ga354/14-1, HU 941/2-5), die Niedersächsisches Ministerium für Wissenschaft und Kultur (11-76251-12-4/12 (ZN2938)) and by the Polish National Science Centre (UMO-2015/19/B/NZ1/00332)

Competing interest: The authors report no competing interests.

For Review Only

References

Al-Robaiy S, Eschrich K. Rat muscle fructose-1,6-bisphosphatase: cloning of the cDNA, expression of the recombinant enzyme, and expression analysis in different tissues. Biol Chem 1999; 380(9): 1079-85.

Barciszewski J, Wisniewski J, Kolodziejczyk R, Jaskolski M, Rakus D, Dzugaj A. T-to-R switch of muscle fructose-1,6-bisphosphatase involves fundamental changes of secondary and quaternary structure. Acta Crystallogr D Struct Biol 2016; 72(Pt 4): 536-50.

Dewulf JP, Wiame E, Dorboz I, Elmaleh-Berges M, Imbard A, Dumitriu D*, et al.* SLC13A3 variants cause acute reversible leukoencephalopathy and alpha-ketoglutarate accumulation. Ann Neurol 2019; 85(3): 385-95.

Dreha-Kulaczewski SF, Brockmann K, Henneke M, Dechent P, Wilken B, Gartner J*, et al.* Assessment of myelination in hypomyelinating disorders by quantitative MRI. J Magn Reson Imaging 2012; 36(6): 1329-38.

Duda P, Janczara J, McCubrey JA, Gizak A, Rakus D. The Reverse Warburg Effect is Associated with Fbp2-Dependent Hif1alpha Regulation in Cancer Cells Stimulated by Fibroblasts. Cells 2020; 9(1).

Frahm J, Bruhn H, Gyngell ML, Merboldt KD, Hanicke W, Sauter R. Localized high-resolution proton NMR spectroscopy using stimulated echoes: initial applications to human brain in vivo. Magn Reson Med 1989; 9(1): 79-93.

Fregno I, Fasana E, Bergmann TJ, Raimondi A, Loi M, Solda T*, et al.* ER-to-lysosome-associated degradation of proteasome-resistant ATZ polymers occurs via receptor-mediated vesicular transport. EMBO J 2018; 37(17).

Gizak A, Dzugaj A. FBPase is in the nuclei of cardiomyocytes. FEBS Lett 2003; 539(1-3): 51-5.

Gizak A, Maciaszczyk-Dziubinska E, Jurowicz M, Rakus D. Muscle FBPase is targeted to nucleus by its 203KKKGK207 sequence. Proteins 2009; 77(2): 262-7.

Gizak A, McCubrey JA, Rakus D. Cell-to-cell lactate shuttle operates in heart and is important in age-related heart failure. Aging (Albany NY) 2020; 12(4): 3388-406.

Gizak A, Pirog M, Rakus D. Muscle FBPase binds to cardiomyocyte mitochondria under glycogen synthase kinase-3 inhibition or elevation of cellular Ca2+ level. FEBS Lett 2012; 586(1): 13-9.

Gizak A, Wrobel E, Moraczewski J, Dzugaj A. Changes in subcellular localization of fructose 1,6-bisphosphatase during differentiation of isolated muscle satellite cells. FEBS Lett 2006; 580(17): 4042-6.

Gurgel-Giannetti J, Lynch DS, Paiva ARB, Lucato LT, Yamamoto G, Thomsen C*, et al.* A novel complex neurological phenotype due to a homozygous mutation in FDX2. Brain 2018; 141(8): 2289-98.

Harris JJ, Attwell D. The energetics of CNS white matter. J Neurosci 2012; 32(1): 356-71.

Helms G, Dathe H, Dechent P. Quantitative FLASH MRI at 3T using a rational approximation of the Ernst equation. Magn Reson Med 2008a; 59(3): 667-72.

Helms G, Dathe H, Dechent P. Modeling the influence of TR and excitation flip angle on the magnetization transfer ratio (MTR) in human brain obtained from 3D spoiled gradient echo MRI. Magn Reson Med 2010; 64(1): 177-85.

Helms G, Dathe H, Kallenberg K, Dechent P. High-resolution maps of magnetization transfer with inherent correction for RF inhomogeneity and T1 relaxation obtained from 3D FLASH MRI. Magn Reson Med 2008b; 60(6): 1396-407.

Houck SA, Ren HY, Madden VJ, Bonner JN, Conlin MP, Janovick JA*, et al.* Quality control autophagy degrades soluble ERAD-resistant conformers of the misfolded membrane protein GnRHR. Mol Cell 2014; 54(1): 166-79.

Huangyang P, Li F, Lee P, Nissim I, Weljie AM, Mancuso A*, et al.* Fructose-1,6-Bisphosphatase 2 Inhibits Sarcoma Progression by Restraining Mitochondrial Biogenesis. Cell Metab 2020; 31(1): 174-88 e7.

Huangyang P, Simon MC. Hidden features: exploring the non-canonical functions of metabolic enzymes. Dis Model Mech 2018; 11(8).

Li H, Handsaker B, Wysoker A, Fennell T, Ruan J, Homer N*, et al.* The Sequence Alignment/Map format and SAMtools. Bioinformatics 2009; 25(16): 2078-9.

Lopez-Hernandez T, Ridder MC, Montolio M, Capdevila-Nortes X, Polder E, Sirisi S*, et al.* Mutant GlialCAM causes megalencephalic leukoencephalopathy with subcortical cysts, benign familial macrocephaly, and macrocephaly with retardation and autism. Am J Hum Genet 2011; 88(4): 422-32.
Mamczur P, Borsuk B, Paszko J, Sas Z, Mozrzymas J, Wisniewski JR*, et al.* Astrocyte-neuron crosstalk regulates the expression and subcellular localization of carbohydrate metabolism enzymes. Glia 2015; 63(2): 328-40.
Mamczur P, Sok AJ, Rzechonek A, Rakus D. Cell cycle-dependent expression and subcellular localization of fructose 1,6-bisphosphatase. Histochem Cell Biol 2012; 137(1): 121-36.
McKenna A, Hanna M, Banks E, Sivachenko A, Cibulskis K, Kernytsky A*, et al.* The Genome Analysis Toolkit: a MapReduce framework for analyzing next-generation DNA sequencing data. Genome Res 2010; 20(9): 1297-303.
Okur V, Cho MT, van Wijk R, van Oirschot B, Picker J, Coury SA*, et al.* De novo variants in HK1 associated with neurodevelopmental abnormalities and visual impairment. Eur J Hum Genet 2019; 27(7): 1081-9.
Pouwels PJ, Brockmann K, Kruse B, Wilken B, Wick M, Hanefeld F*, et al.* Regional age dependence of human brain metabolites from infancy to adulthood as detected by quantitative localized proton MRS. Pediatr Res 1999; 46(4): 474-85.
Provencher SW. Estimation of metabolite concentrations from localized in vivo proton NMR spectra. Magn Reson Med 1993; 30(6): 672-9.
Rimmer A, Phan H, Mathieson I, Iqbal Z, Twigg SRF, Consortium WGS*, et al.* Integrating mapping-, assembly- and haplotype-based approaches for calling variants in clinical sequencing applications. Nat Genet 2014; 46(8): 912-8.
Schneider CA, Rasband WS, Eliceiri KW. NIH Image to ImageJ: 25 years of image analysis. Nat Methods 2012; 9(7): 671-5.
Sobreira N, Schiettecatte F, Valle D, Hamosh A. GeneMatcher: a matching tool for connecting investigators with an interest in the same gene. Hum Mutat 2015; 36(10): 928-30.
van der Knaap MS, Bugiani M. Leukodystrophies: a proposed classification system based on pathological changes and pathogenetic mechanisms. Acta Neuropathol 2017; 134(3): 351-82.
van der Knaap MSV, J. Magnetic Resonance of Myelination and Myelin Disorders. 3rd ed. Berlin Heidelberg; 2005.
Wisniewski J, Pirog M, Holubowicz R, Dobryszycki P, McCubrey JA, Rakus D*, et al.* Dimeric and tetrameric forms of muscle fructose-1,6-bisphosphatase play different roles in the cell. Oncotarget 2017; 8(70): 115420-33.
Yan H, Helman G, Murthy SE, Ji H, Crawford J, Kubisiak T*, et al.* Heterozygous Variants in the Mechanosensitive Ion Channel TMEM63A Result in Transient Hypomyelination during Infancy. Am J Hum Genet 2019; 105(5): 996-1004.
Yip J, Geng X, Shen J, Ding Y. Cerebral Gluconeogenesis and Diseases. Front Pharmacol 2016; 7: 521.
Zarzycki M, Kolodziejczyk R, Maciaszczyk-Dziubinska E, Wysocki R, Jaskolski M, Dzugaj A. Structure of E69Q mutant of human muscle fructose-1,6-bisphosphatase. Acta Crystallogr D Biol Crystallogr 2011; 67(Pt 12): 1028-34.

For Review Only

Figure legends

Figure 1: Pedigree and segregation analysis of the affected Family. Nomenclature is according to GenBank accession number NM_003837.2. Case reports on III.2, III.5 and IV.1 can be found in the results section. I.2, female, was born in 1948 and developed epilepsy and schizophrenia during her youth. She has never learnt a profession and lives in a shelter home. Her two children, II.2 II.4, grew up in an orphanage in the GDR because she was incapable of looking after her. No information is available for the first 5 years of their life. Patient II2 developed severe anorexia as a teenager, which currently at age 47 is still present and prevents her from working in her profession as a nurse. II.4, developed epilepsy at age 4 years, and still now requires anticonvulsive medication at age 46. She works as a cleaner. III.3, aged 26 years, has a history of psychosis and drug addiction. He refused cerebral MRI investigation. III.4 developed epilepsy at age 9 years following a presumed viral infection with high fever. Development up to this point of time was normal with no signs of a metabolic crisis. She was treated with oxcarbazepine but continued to have two brief (2-3 minutes) focal seizures a month until age 13 years. At last presentation at age 14.5 years she was seizure free and visiting a regular school. IV.2, now aged 6 years, has mildly delayed speech development and an attention deficit hyperactivity disorder. Family members I.1, II.1, II.3, II5 and III.1 have no known neurological or psychiatric disorders.

Figure 2: MR imaging of the family members. A-K) Axial MR images of the patients with the remitting leukodystrophy. A-C) T2 weighted images of patient III.2 at age 14 months, 22 months and 20 years. D-G) T2 weighted images of patient III.5 at age 11 months, 21 months, 32 months and 24 years. H-K) T2 weighted images of patient IV.1 at age 13, 19, 25 and 29 months. L-O) ADC of patient IV.1 at corresponding time points. P, Q) T2 weighted images of patient III.4 at age 10 (P) and 15 years (Q) with residual WM lesions, pronounced frontally and in the genu corpus callosum. R) FLAIR images of patient II.2 at age 38 years revealing frontally pronounced residual WM lesion. S) FLAIR images of patient II.4 at age 46 years with similar residual pattern and global atrophy. T) T2 weighted images of patient IV.2 at age 4 years. Of note are the subtle WM hyperintensities in genu and splenium, comparable to patient III.4, II.2, II.4, although no metabolic crisis occurred.

Figure 3: Quantitative MR spectroscopy and Myelin sensitive imaging. A) Lactate levels (in mM) in patients III.2, III.5 and IV.1 at different time points. Over time lactate returned to normal ranges (≤ 1mM) in the two follow up studies. B – D) Overlay of color-coded axial MTsat maps onto corresponding T1 weighted images of patient IV.1. At age 13 months (B)

For Review Only

blue color-coded white matter areas depict myelin deficit; at 19 months (C) extension of blue-gray color-coded WM regions and appearance of light blue-coded WM frontally representing very low MT values (close to CSF) reveal ongoing demyelination; at 29 months (D) appearance of gray-red-orange-yellow color-coded WM areas illustrate remyelination. E) Overlay of color-coded axial MTsat map onto corresponding T1 weighted image of a healthy control age 18m and the color scale encoding the MTsat values. Note the predominance of red-orange coded MTsat values and transition to yellow coded WM pointing to sufficient myelination.

Figure 4: Comparison of kinetics and stability of the FBP2 variants.

A) Comparison of V115M-FBP2 and WT-FBP2 kinetic parameters, and B) thermal stability of the mutant protein. In the panel B), solid lines represent mean melting curves from three independent measurements, and dashed lines indicate mean melting temperatures. C) Enzymatic activity of V115M-FBP2 and WT-FBP2 after prolonged incubation at 37°C. The activity was normalized to mean activity at t=0 for the respective FBP2 form.

Figure 5: Subcellular localization of the FBP2 variants.

A) In healthy control fibroblasts (HCF), FBP2 is homogeneously distributed in whole cytoplasm and nuclei (arrows), as determined by immunocytochemistry. In fibroblasts derived from patient III.5 (Leucodystrophy Fibroblasts, LeuF), nuclei (arrows) are empty, and the enzyme accumulates only in their vicinity, not in cellular protrusions. B) GSK3β inhibition results in accumulation of FBP2-related immunofluorescent signal with mitochondria in HCF, but not in LeuF. C) In control conditions, FITC-labeled V115M-FBP2 does not co-localize with mitochondria of HCF, while WT-FBP2 does (arrows). D) Mitochondrial network in LeuF is much less developed and membrane of the organelles is less polarized than in HCF. Total as well as mitochondrial production of reactive oxygen species (ROS) in LeuF is higher than in HCF.

Figure 6: Expression and degradation of the FBP2 variants.

A) Total fluorescent signal associated with polyclonal antibodies against FBP is stronger in Leucodystrophy Fibroblasts (LeuF) than in healthy control fibroblasts (HCF). B) Similarly, the signal for total FBP2 mRNA is much stronger in LeuF than in HCF. V115M-FBP2 mRNA is not detected in HCF. C) In HCF, FITC-labeled WT-FBP2 hardly co-localizes with ER while V115M-FBP2 highly co-localizes with ER in control conditions, but not after ERAD inhibition. D) Total fluorescent signal associated with K48 polyubiquitination is slightly but significantly

For Review Only

stronger in LeuF than in HCF. E) The majority of FBP-related granules co-localize with proteasome subunit PSMC6. F) LAMP-1 level is over 2.5-times higher in LeuF than in HCF.

Supplementary Figure 1: Confirmation of heterozygous *FBP2* variant. A) Sanger sequencing chromatogram of the *FBP2* mutation side is shown for each investigated family member with different mutation status wild type (WT) and heterozygous mutated (het). The position of the affected *FBP2* nucleotide triplet coding for p.V115M is highlighted by a box. Nomenclature is according to GenBank accession number NM_003837.2 and NP_003828.2.

Supplementary Figure 2: Multiple Alignment and stereo representation of Val115 position in the FBP2 structure. A) The multiple alignment shows that the Val115 position is strongly conserved with hydrophobic residues for FBP in vertebrates, but also insects, plant, whereas there is an Ala as an even smaller hydrophobic side chain with Aspargillus. B) Stereo view „crossed eye“ of FBP2 subunit A with structural elements in schematic representation. Val115 space filling in red, surrounding hydrophobic residues Val94, Tyr140, Ala153, and the carbon backbone of Lys142 side chain in green, subunit C in dark blue. The figure was drawn with WebLab-Viewer (Molecular Simulations Inc., Burlington, MA, USA), using the X-ray structure of human FBP2 (pdb code 5et7).

For Review Only

Supplementary Table 1: Basic WES information and output data. Overview of coverage, mapping information and homozygosity for each WES sample. Consanguinity can be estimated using % of rare hom variants (3 – 6%) and autosomal runs of homozygosity (> 200 – 300 mb). Cov, coverage; hom, homozygosity

Supplementary Table 2: Detected filtered common variants. Detailed variant information with localization, substitution and occurrence within the family. Het, heterozygous

Supplementary Table 3: Detected filtered common variants. Detailed variant information corresponding bioinformatic prediction on the functional effect, conservation and occurrence in ExAC. Variant classification for each tool was obtained using dbNSFP version 3.4.

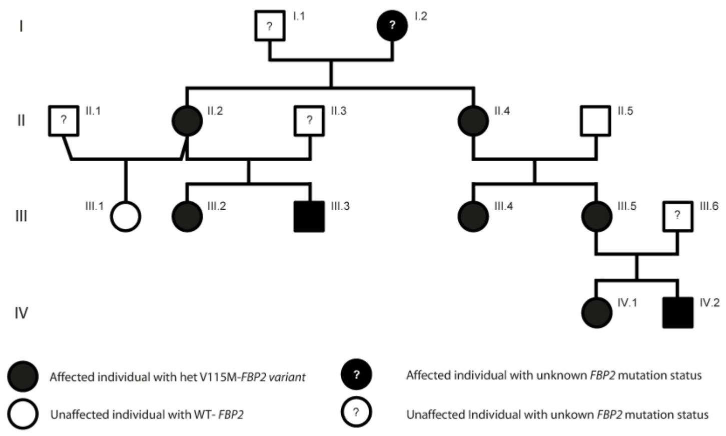

Figure 1

Figure 1

210x297mm (600 x 600 DPI)

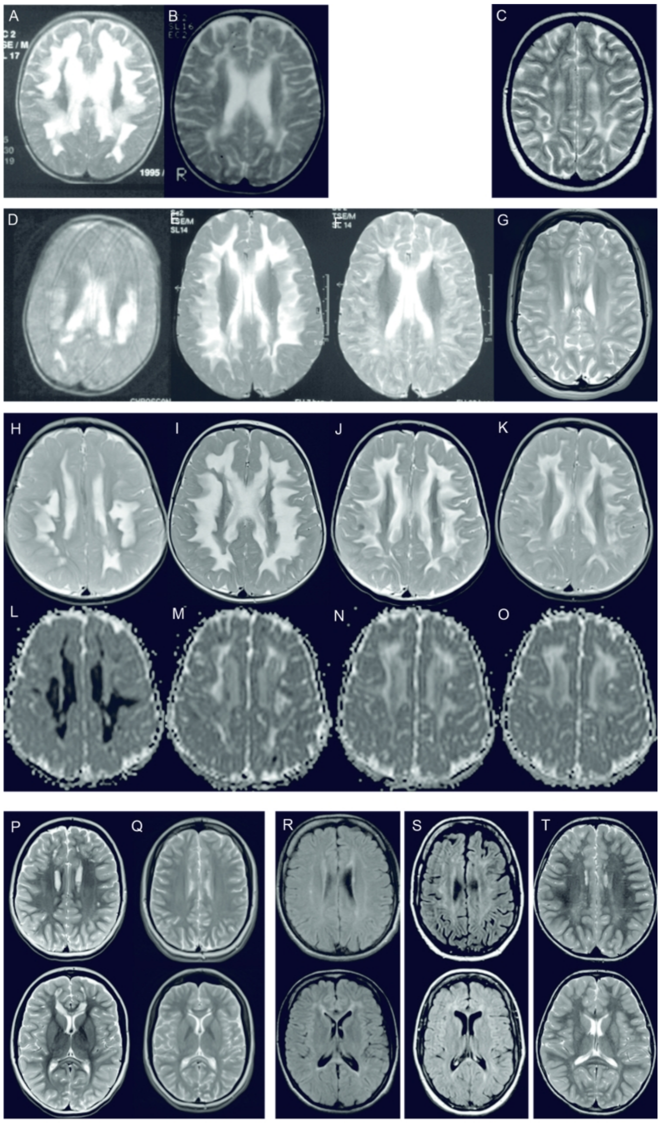

Figure 2

Figure 2

135x248mm (300 x 300 DPI)

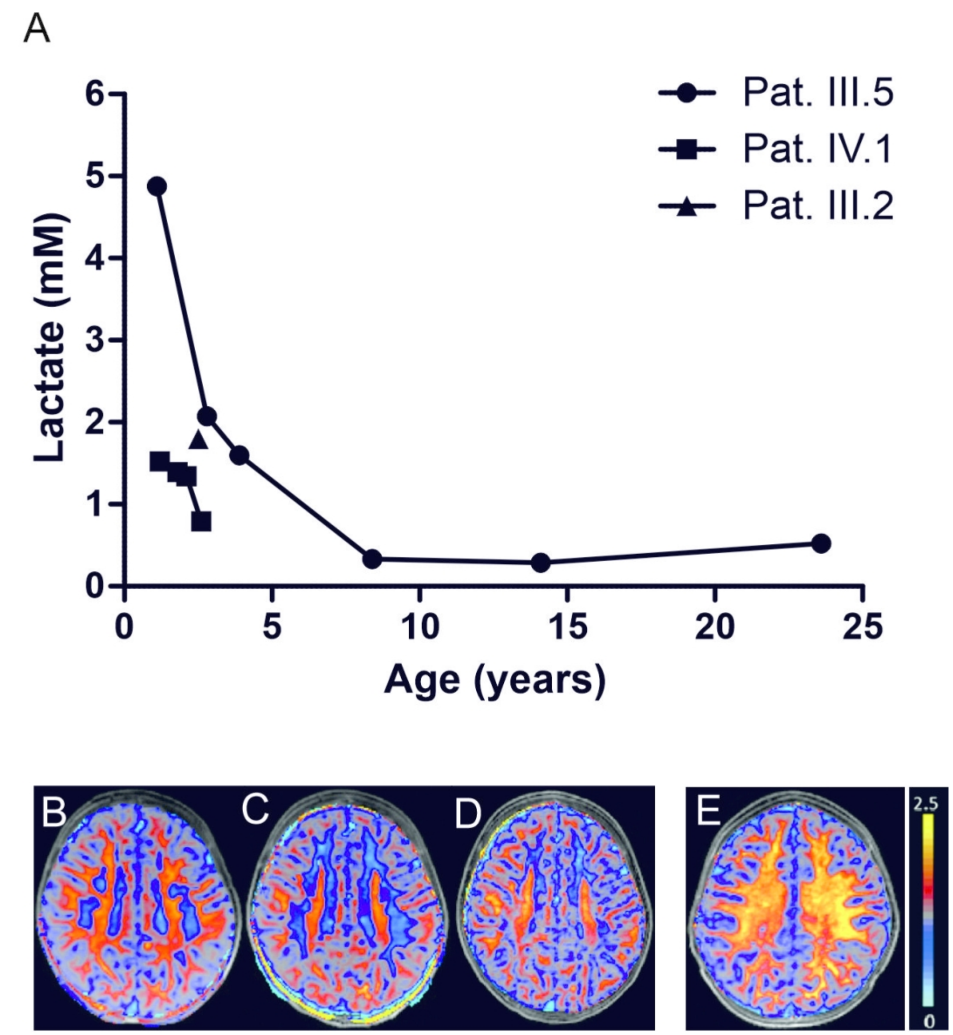

Figure 3

Figure 3

111x141mm (300 x 300 DPI)

A)

FBP2	k_{cat} [s^{-1}]	K_s [μM]	K_{is} [μM]	n, F-1,6-BP	IC_{50}, AMP [μM]	n, AMP
WT	20.96±2.52	3.66±0.08	111±2	1.61±0.036	0.592±0.01	2.04±0.0515
V115M	11.47±0.22	5.39±0.19	167.7±8.55	1.32±0.03	1.23±0.14	1.187±0.099

Data are presented as mean±SD

k_{cat} – catalytic constant, K_s – substrate constant, K_{is} – substrate inhibition constant, n – Hill coefficient, IC_{50} – half inhibition constant

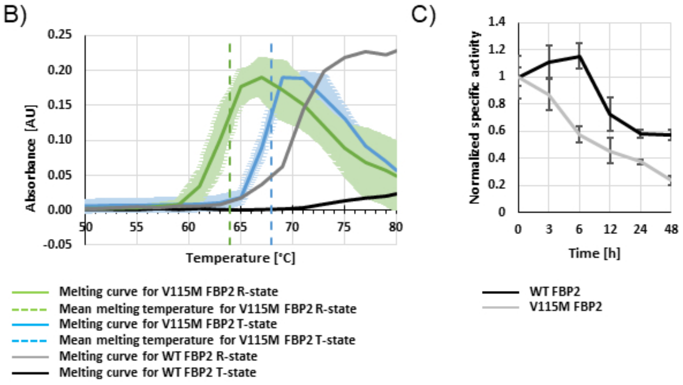

Figure 4

Figure 4

190x275mm (96 x 96 DPI)

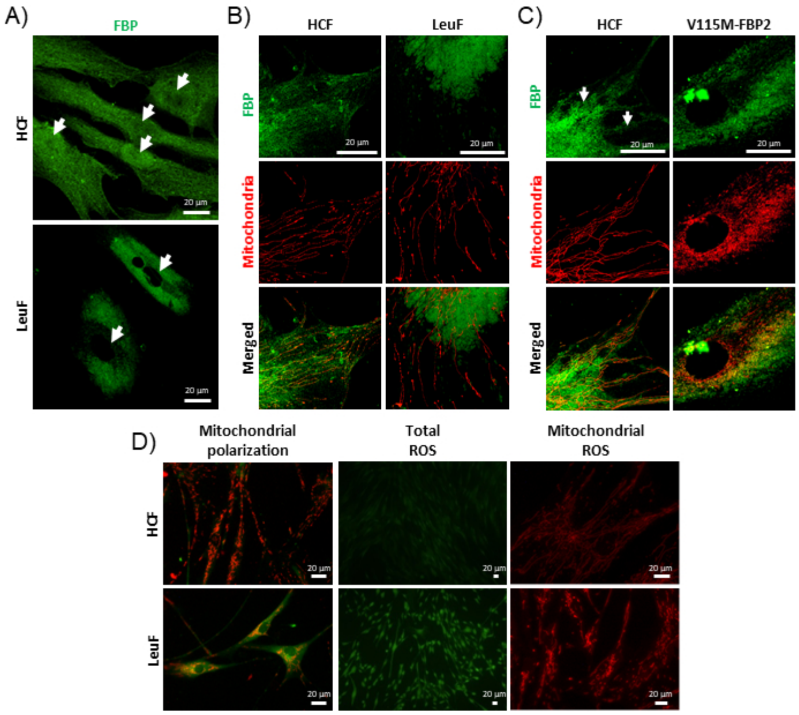

Figure 5

Figure 5

190x275mm (96 x 96 DPI)

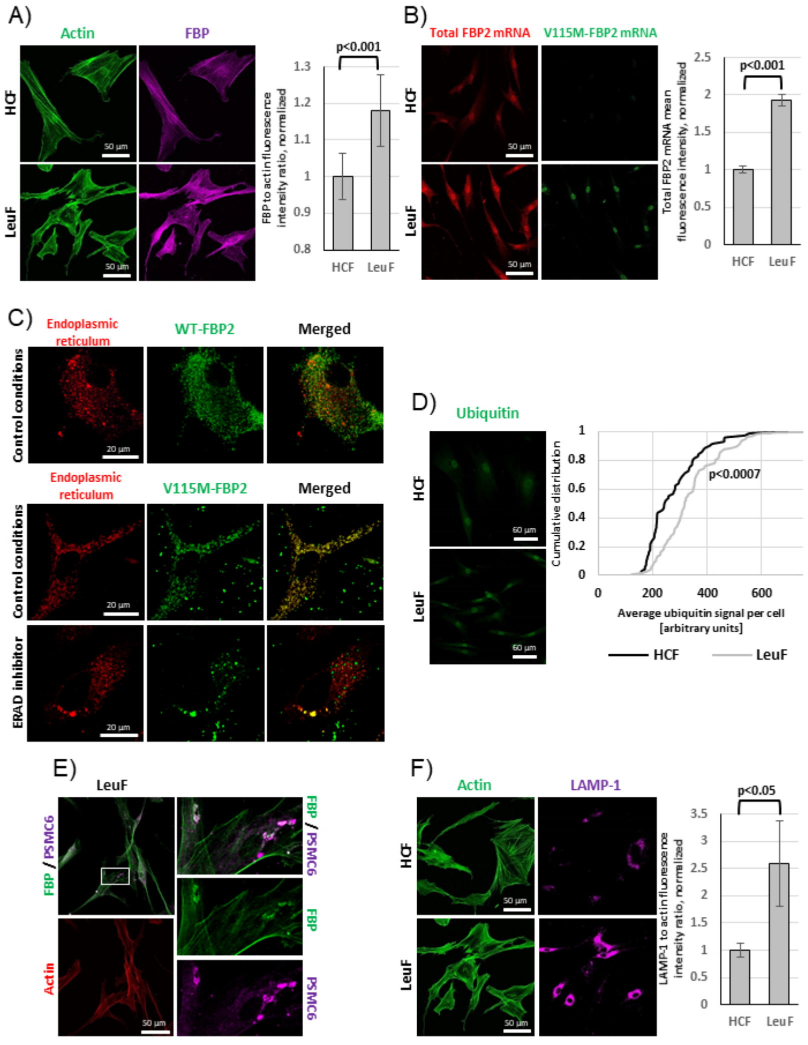

Figure 6

Figure 6

190x275mm (96 x 96 DPI)

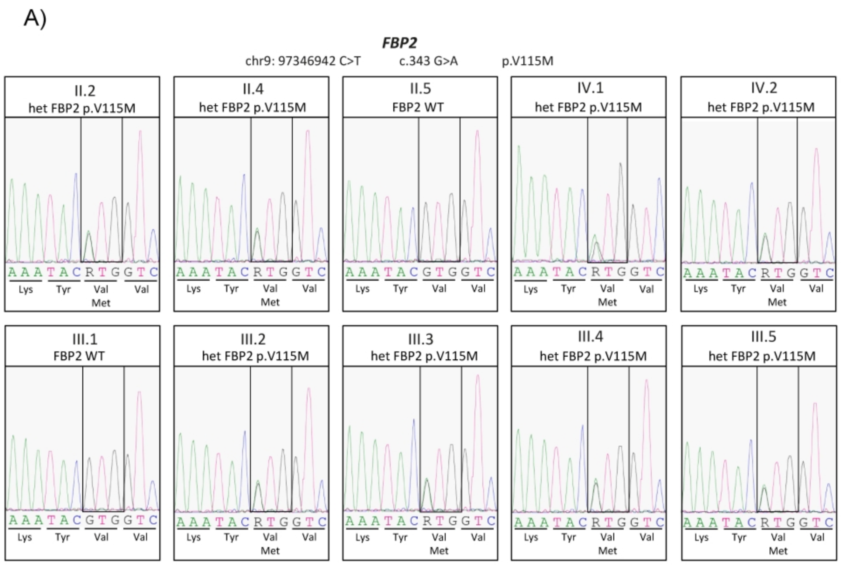

Supplementary Figure 1

210x297mm (600 x 600 DPI)

A)

```
                              130       140       150       160       170       180
                                |         |         |         |         |         |
                                                                               *V115M
F16P2_HUMAN               IAGSVNVTGDEVKKLDVLSNSLVINMVQSSYSTCVLVSEENKDAIITAKEKRGKYVVCFD
Bos_taurus                IAGSVNVTGDEVKKLDVLSNALVINMLQSSYSTCVLVSEENKEAIITSKEKRGKYVVCFD
Gallus_gallus_FBP2        IAGTVNVTGDEVKKLDVLSNSLVINMLQSSYSTCVLVTEENKEALITPKEKRGKYVVCFD
Xenopus_tropicalis        ISGSVNVTGDEVKKLDVLSNDLVINMLKSSYGTCALVSEENKEVIIIPKEMQGKYVVCFD
Danio_rerio               IAGQVNVTGDEQKKLDVLSNDLIINLLQASYGTCLMVSEENKDAIYTPAEKRGKYIVCFD
human_FBP1                IAGSTNVTGDQVKKLDVLSNDLVMNMLKSSFATCVLVSEEDKHAIIVEPEKRGKYVVCFD
Anopheles_Gambiae_FBP     ISGDTNVQGEQVKKLDVLSNEIFINMLKSSYATCLLVSEENDNVIEIETDKRGKYVVSFD
Glycine_max_FBP1          VQGAVNVQGEDQKKLDVVSNEVFSNCLRSSGRTGIIASEEEDVPVAVEESYSGNYIVVFD
Aspargillus_niger_FBP     LAGSSNTTGDDQKKLDVIGNDIFISAMKGSGKCRILVSEEEEEAIVFDEHPNARYAVVCD
                          : *  *. *:: *****:.* :. . ::.*     :.:**:.  :       ..* *  *
Prim.cons.                IAGSVNVTGDEVKKLDVLSNDLVINML2SSYSTCVLVSEENKEAIIT2KEKRGKYVVCFD

                              190       200       210       220       230       240
                                |         |         |         |         |         |
F16P2_HUMAN               PLDGSSNIDCLASIGTIFAIYRKTSE--------------DEPSEKDALQCGRNIVAAGY
Bos_taurus                PLDGSSNIDCLASIGTIFAIYRKTSE--------------DEPSEKDALQPGRNIVAAGY
Gallus_gallus_FBP2        PLDGSSNIDCLAPIGTIFAIYKKETD--------------DEPSEKDALQPGRKIVAAGY
Xenopus_tropicalis        PLDGSSNIDCLASIGTIFAIYRKTTD--------------TEPCEQDALQPGRNIVAAGY
Danio_rerio               PLDGSSNIDCLAPIGTIFAIYKRISD--------------GEPSEKDALQPGNQIVCAGY
human_FBP1                PLDGSSNIDCLVSVGTIFGIYRKKST--------------DEPSEKDALQPGRNLVAAGY
Anopheles_Gambiae_FBP     PLDGSSNIDCLVSIGSIFAITKQVKEN-------------TTPSEQDALQPGNKIVAAGY
Glycine_max_FBP1          PLDGSSNIDAAVSTGSIFGIYSPNDECLADIDDDPTLDTTEQRCIVNVCQPGSNLLAAGY
Aspargillus_niger_FBP     PIDGSSNLDAGVSVGTIFGIFKLPDEVLGPN---------NKVTAQDLLRAGTEMVASGF
                          *:*****:*.  .. *:**.*                         :  : * :::.:*:
Prim.cons.                PLDGSSNIDCLASIGTIFAIY2KTSE3L222DDDPTLDTTDEPSEKDALQPGRNIVAAGY
```

B)

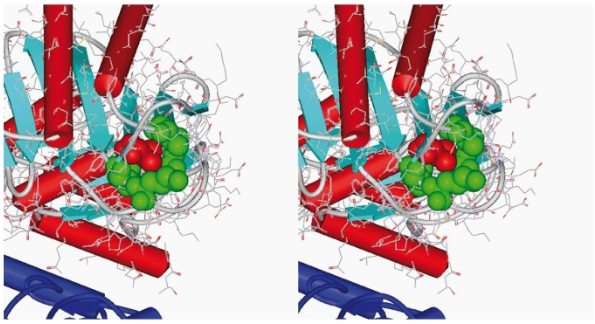

Supplementary figure 2

210x297mm (600 x 600 DPI)

Sample	Varbank Pipeline	read length	Mean Cov	Cov 30x	Total reads	Unique mapped reads [%]	autosomal Runs of Homozygosity [Mb]	% of rare hom variants	Gender
II.2	v 3.5	101	95	86.0	122789264	79.00	15	0.1	Female
II.4	v 3.5	101	83	83.1	107980136	78.85	21	0.4	Female
III.2	v 3.4	76	75	81.9	132206850	74.67	8	0.1	Female
III.4	v 3.4	76	66	72.9	115661122	74.87	7	0.1	Female
III.5	v 3.5	101	74	76.9	100459852	82.61	34	0.0	Female
IV.1	v 3.5	101	73	80.7	106791788	87.73	14	0.0	Female

Supplementary Table 1

For Review Only

Gene	Chr	Position	Ref	Mut	Transcript ID	cDNA	Protein	Sample II.2	II.4	III.2	III.4	III.5	IV.1
CCT6A	7	56055733	T	C	NM_001762.3	c.446T>C	p.Ile149Thr	het	het	het	het	het	het
FBP2	9	94584660	C	T	NM_003837.3	c.343G>A	p.Val115Met	het	het	het	het	het	het

Supplementary Table 2

For Review Only

	Tool	Range	*CCT6A* NM_001762.3 p.I149T	Prediction	*FBP2* NM_003837.3 p.V115M	Prediction
	Median Rank Score	0 - 1	0.48		0.65	
FUNCTIONAL PREDICTION SCORES	SIFT	0 - 1	0.176	Tolerated	0.011	**Damaging**
	PolyPhen2	0 - 1	0.009	Benign	0.999	**Damaging**
	LRT	0 - 1	0.000290	Neutral	0.000	**Damaging**
	MutTaster	0 - 1	0.998	**Damaging**	1.000	**Damaging**
	MutAssessor	-5.545 - 5.975	1.3	Low functional impact	2.535	**Medium functional impact**
	FATHMM	-16.13 - 10.64	-1.15	Tolerated	-0.74	Tolerated
	PROVEAN	-14 - 14	-2.39	Neutral	-2.26	Neutral
	VEST3	0 - 1	0.195	Tolerated	0.638	**Damaging**
	MetaSVM	-2 - 3	-0.7441	Tolerated	0.1394	**Damaging**
	MetaLR	0 - 1	0.2964	Tolerated	0.5455	**Damaging**
	CADD	-7.5350 - 35.7885	1.3315	Neutral	5.6513	**Damaging**
	DANN	0 - 1	0.8208	Neutral	0.9987	**Damaging**
	FitCons	0 - 1	0.7354	**Functional important**	0.5731	Less Functional important
CONSERVATION SCORES	GERP	-12.3 – 6.17	2.39	**High conserved**	4.08	**High conserved**
	PhastCons	0 - 1	0.996	**High conserved**	0.980	**High conserved**
	PHYLOP20	-13.282 to 1.199	0.824	**High conserved**	0.876	**High conserved**

Supplementary Table 3

For Review Only
