# Supplementary material for: A novel remitting leukodystrophy associated with a variant in FBP2
Source: Brain Commun. 2021 Mar 11;3(2):fcab036. doi: 10.1093/braincomms/fcab036 (PMC8097510; doi:10.1093/braincomms/fcab036)
Supplement: fcab036_Supplementary_Data [file fcab036_supplementary_data.zip › Review_files.zip!/Revision 1 - Decision Letter.pdf]

Revision 1 – Decision Letter

18-Jan-2021

Dear Dr. Huppke,

It is a pleasure to accept your manuscript entitled "A novel remitting leukodystrophy associated with a variant in FBP2" for publication in Brain Communications.

The next step is for your paper to go through “First Look”. We will contact you again shortly if any final files or formatting changes are required for publication or if we have any queries.

If no changes are needed, your submission will be transferred to our Production Department and you will hear from the Production Editor in due course.

Video abstracts - Brain Communications encourages authors to provide a video abstract to accompany their article. These are hosted on the Brain/Brain Communications YouTube channel. If you are interested in producing a 5-min video abstract to promote your article, then please contact the Editorial Office (brcoms.editorialoffice@oup.com) for further details and guidelines.

Thank you very much for your contribution. On behalf of the Editors of Brain Communications, we look forward to your future contributions to the Journal.

Yours sincerely,

Dr José Brás
Associate Editor
Brain Communications
